# Paracrine Factors Released by Stem Cells of Mesenchymal Origin and their Effects in Cardiovascular Disease: A Systematic Review of Pre-clinical Studies

**DOI:** 10.1007/s12015-022-10429-6

**Published:** 2022-07-28

**Authors:** Nishani S. Mabotuwana, Lavinia Rech, Joyce Lim, Sean A. Hardy, Lucy A. Murtha, Peter P. Rainer, Andrew J. Boyle

**Affiliations:** 1 College of Health, Medicine and Wellbeing, The University of Newcastle, Newcastle, NSW Australia; 2 Hunter Medical Research Institute, Lot 1, Kookaburra Circuit, Newcastle, NSW 2305 Australia; 3 Department of Internal Medicine, Division of Cardiology, Medical University of Graz, Graz, Austria; 4 Department of Cardiac Surgery, Boston Children’s Hospital and Harvard Medical School, Boston, MA USA; 5 Department of Cardiovascular Medicine, John Hunter Hospital, Newcastle, NSW Australia; 6 BioTechMed Graz, Graz, Austria

**Keywords:** Mesenchymal stem cell, Paracrine, Secreted, Myocardial ischemia, Cardiac repair, Cardiac regeneration

## Abstract

Mesenchymal stem cell (MSC) therapy has gained significant traction in the context of cardiovascular repair, and have been proposed to exert their regenerative effects via the secretion of paracrine factors. In this systematic review, we examined the literature and consolidated available evidence for the “paracrine hypothesis”. Two Ovid SP databases were searched using a strategy encompassing paracrine mediated MSC therapy in the context of ischemic heart disease. This yielded 86 articles which met the selection criteria for inclusion in this study. We found that the MSCs utilized in these articles were primarily derived from bone marrow, cardiac tissue, and adipose tissue. We identified 234 individual protective factors across these studies, including VEGF, HGF, and FGF2; which are proposed to exert their effects in a paracrine manner. The data collated in this systematic review identifies secreted paracrine factors that could decrease apoptosis, and increase angiogenesis, cell proliferation, and cell viability. These included studies have also demonstrated that the administration of MSCs and indirectly, their secreted factors can reduce infarct size, and improve left ventricular ejection fraction, contractility, compliance, and vessel density. Furthering our understanding of the way these factors mediate repair could lead to the identification of therapeutic targets for cardiac regeneration.

**Graphical abstract:**

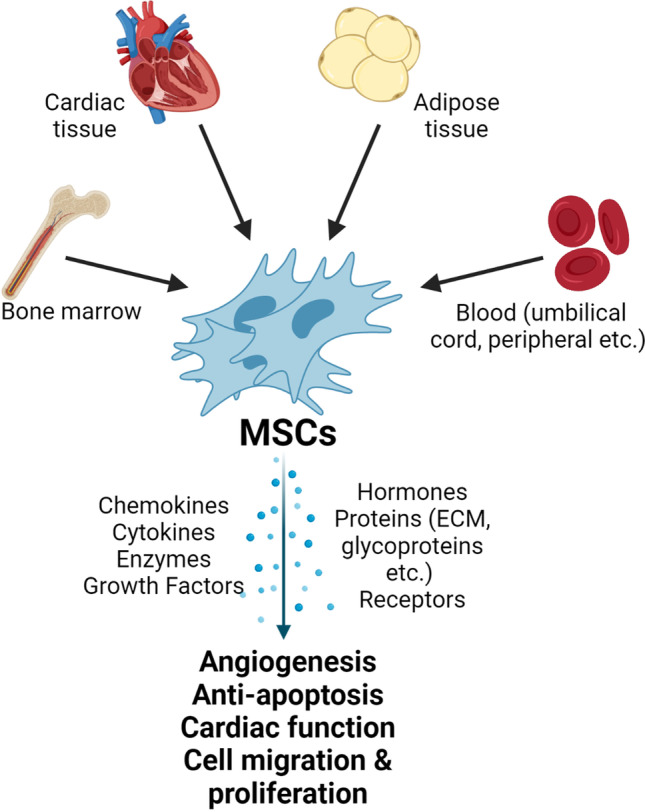

**Supplementary Information:**

The online version contains supplementary material available at 10.1007/s12015-022-10429-6.

## Introduction

The adult mammalian heart exhibits limited capacity for cellular regeneration, thus injuries causing myocyte loss such as a myocardial infarction (MI) result in the activation of pro-fibrotic pathways that initiate healing following a cardiac insult but also lead to irreversible scarring. Long-term activation of these pathways results in ventricular stiffness, contractile dysfunction, and cellular hypertrophy and apoptosis. Ultimately, these pathological changes severely impair physiological functioning of the heart, and lead to the irreversible development of heart failure, for which therapeutic options are currently limited.

Stem cell therapy has emerged as a promising approach to repair the damaged myocardium, with the aim of providing the infarcted heart with an exogenous supply of regenerative elements to promote cytoprotection, vascularization, or cardiomyogenesis [1]. In particular, there has been a focus on cells of mesenchymal origin (mesenchymal stem cells – MSCs), including bone marrow derived MSCs (BM-MSCs) and cardiac progenitor cells (CPCs). Several populations of resident CPCs have been identified including c-kit^+^, Sca-1^+^, Islet 1^+^, and cardiospheres, all of which have promoted cardiac repair to varying degrees [2, 3]. These cell populations are cardiac lineage committed, and may offer a significant advantage when compared to their counterparts. However, given their limited numbers in the heart, they do not adequately promote cardiac repair following an acute injury independently. Nonetheless, treatment with BM-MSCs [4] and CPCs [5] in pre-clinical studies has resulted in improvements in left ventricular ejection fraction (LVEF), contractility, increased angiogenesis, and reduced infarct size. In vitro, these cells have demonstrated a capacity to differentiate into cardiomyocytes and vascular endothelial cells [5, 6], but there is no clear evidence of differentiation in vivo either pre-clinically or clinically [7]. Furthermore, studies have consistently shown that implanted BM-MSCs [8] and CPCs [9] engraft efficiently or do not survive longer than 3 weeks post-injection, suggesting that differentiation is unlikely to be the primary mechanism driving the observed improvements in cardiac outcomes. The secretion of soluble paracrine factors has been proposed as an alternative mechanism and this is termed the “paracrine hypothesis”.

Stem cells condition culture media by producing and secreting a range of cytokines, chemokines, and growth factors in their culture media. In support of the paracrine hypothesis, numerous studies have demonstrated that conditioned media alone has a similar protective effect to whole cell therapy in vitro [10–13] and in vivo [12], including promotion of cell survival and proliferation, immunomodulation, cardiac remodelling, neovascularization, and activation of resident CPC populations [14–16]. Some soluble factors known to be produced and released by adult stem cells include VEGF, FGF2, HGF, IGF1, IL1β, IL15, PDGF, and SDF1, [11, 12, 17]. The available literature has also identified the release of exosomes and extracellular vesicles by stem cells. The study of these vesicles is multifaceted in its nature given the complexity of characteristics, functions, and biological processes associated with them. Given they are an additional cargo packaging a range of bioactive factors such miRNAs, mRNA molecules, peptides, proteins, cytokine, and lipids, they would warrant an in depth analysis of their own right [18, 19]. For this reason, and in the interest of presenting a concise body of work, we have focused exclusively on factors shown to be directly released by stem cells of mesenchymal origin.

Despite stem cells being capable of exerting cardioprotective effects as a whole, the molecular mechanisms underpinning the release and action of individual factors vary. Consolidating factors known to be directly secreted by MSCs thus far would be beneficial as their application may circumvent the need for whole cell therapy, which possesses numerous problems including the cost and time to grow and deliver cells, donor matching, immune rejection, and the ethical and legal concerns associated with each of the potential cell types. Studies are already investigating the targeted delivery of specific factors such as HGF, IL15, and VEGF and have shown some reductions in scar size, and attenuated signs of cardiac remodelling to a certain extent in pre-clinical models of MI [20, 21]. Whilst promising, it is likely that a combination of factors would more successfully promote cardiac repair following an acute injury and numerous repair mechanisms would need to act in concert to allow recovery.

The aim of this systematic review is to consolidate the existing literature and identify paracrine factors directly released by MSCs, which may improve cardiac healing. Where available, data concerning their functional effects in vitro, in vivo, or ex vivo was extracted. In this review, we have identified a range of stem cells of mesenchymal origin, including MSCs derived from adipose tissue (AD-MSCs, APCs), bone marrow (BM-MSCs), cardiac tissue (CPCs, CSCs), menstrual blood (En-MSCs), placenta (P-MSCs), peripheral blood (PB-MSCs), and umbilical cord blood (UCB-MSCs). Throughout this article, the term MSCs will be broadly used to refer to these cell types as a whole.

## Methods

### Search Strategy

A systematic literature search was conducted using Ovid SP databases (Embase and Medline), and included all relevant publications to the 22 February 2022. The search strategy used for Embase and Medline are outlined in the supplementary information Tables 1 and 2 respectively. Upon completion of the search, duplicate texts were removed, uploaded to Covidence, and the titles and abstracts of the remaining articles examined for relevance to the review topic. Those that did not fit the inclusion criteria were noted, but not analyzed further. PROSPERO systematic review database registration: CRD42019127475. During the full text screening and data extraction process it became clear that the proposed quality assessment tools in our PROSPERO protocol would not be sufficient to investigate the question at hand, and thus we designed a checklist (detailed below) to better address the question at hand.

### Inclusion Criteria

Retrieved texts were screened for relevance based on the inclusion criteria detailed below. Original research articles were included if they met the primary aim of identifying paracrine factors directly released by MSCs which may be capable of mediating improvements in a cardiac context. In vitro studies were included if they: 1) clearly identified the mesenchymal origin of cell type used, 2) identified protective factors released directly by MSCs thought to be behaving in a paracrine manner in the study, and 3) included of appropriate control groups in the study design. Where included studies contained relevant ex vivo or in vivo cardiac models, the reported functional associations of stem cell therapy were additionally summarized. All searches were limited to English-language articles published by 22 February 2022.

### Exclusion Criteria

Review articles, conference proceedings and retracted studies were excluded from this systematic review. This review focuses on identifying paracrine factors directly released by cells of mesenchymal origin. As such, studies which: 1) used cells of non-mesenchymal origin, 2) did not directly demonstrate release of paracrine factors by cell types being investigated, or identified particles such as extracellular vesicles or exosomes, 3) investigated the protective effects of treating MSCs without appropriate controls, or 4) investigated the protective effects of culturing MSCs on biomaterials without appropriate controls were excluded from this review.

### Study Selection

Three investigators (N.S.M., L.R., and J.L.) independently evaluated the titles and abstracts (n = 4443) of the identified articles according to the selection criteria, those articles of potential relevance were allocated to the next stage to be reviewed in full (n = 275). Three investigators (N.S.M., L.R., and A.J.B.) independently undertook full text screening according to the inclusion and exclusion criteria outlined above. In cases of initial disagreement on an article’s eligibility, a decision was rendered following discussion leading to consensus between investigators. Initial agreement between investigators on the eligibility of an article was assessed using percentage agreement and the kappa statistic.

### Data Extraction and Quality Assessment

The following data were extracted from included studies: first author, year of publication, origin of MSCs, phenotyping of MSCs, study design, identified paracrine factors, and method used to identify paracrine factors. In studies where MSCs were treated, transfected, or cultured on biomaterials only data from appropriate control groups were considered for analysis. Data regarding in vitro, ex vivo or in vivo models of cardiac ischemia were additionally extracted. We developed a 9-point checklist (Table 1) to assess the quality of reporting and overall study design.

**Table 1:** 9-point quality assessment checklist

Criteria	0	1	2
Were the aims/objectives of the study clearly stated?	Not stated	Aims/objectives are somewhat clear	Aims/objectives are clearly stated
Were the main outcomes to be measured clearly described in the introduction/ methods?	Not stated	Some outcomes to be measured described	All outcomes to be measured clearly described
Were the main findings clearly described?	Not clearly described	Reported findings are somewhat clear	Reported findings are clearly described
Was the source of stem cells used in the study clearly described?	Type of biological material the stem cells were derived from is unclear	Type of biological material the stem cells were derived from were clearly identified	Type of biological material, gender and species the stem cells were derived from were clearly identified
Were the stem cells used in the study clearly shown to be mesenchymal using either the minimum International Society for Cellular Therapy (ISCT) criteria to identify multipotent human mesenchymal stem cells (MSCs) or other validated markers? ISCT Criteria: 1. Adherence to plastic 2. Multipotent differentiation potential into adipogenic, osteogenic, and chondrogenic lineages 3. Flow cytometry or immunocytochemistry to show that cells are CD105 (SH2), CD73 (SH3), CD90 positive; and CD45, CD34, CD14/CD11b, CD79α/CD19, HLA-DR negative Other validated markers: CD29, CD44, CD49a-f, CD51, CD106, CD166, Stro-1, CD13, CD10, Sca-1/Ly6, CD200, GD2, CD146, c-kit positive	None of the minimum criteria were reported in the current study OR study does not cite previous publications in which characterising was conducted	1 to 3 of the minimum criteria were reported in the current study OR study cites previous publications in which characterising was conducted Where multipotent differentiation potential was assessed, differentiation into 2 lineages shown Where surface markers were characterized, a panel of some positive and negative markers from ISCT guidelines or other validated markers were measured	All 3 of the minimum criteria were reported in the current study and stem cells used in the study are in complete accordance with ISCT guidelines Other validated markers can be included in addition to ISCT markers
Were in vitro experiments conducted at minimum as three independent experiments?	Sample size was not reported, unclear, or experiments conducted in duplicate or less	Experiments conducted as technical replicates, in triplicate at minimum	Experiments conducted as at least 3 independent experiments
Were the number of cells, and passage of cells used in an experiment clearly stated?	Neither the number of cells used nor passage cells were used at were reported	Either the number of cells used, or the passage cells were used at were reported	Both the number of cells used, and the passage cells were used at were reported
Is an appropriate control group present in study?	No appropriate control groups	Study includes some controls	Study is well controlled
Are the statistics used appropriate?	No statistics/ inappropriate statistics	Appropriate statistics used	–

## Results

### Selection of Studies

**Fig. 1:**
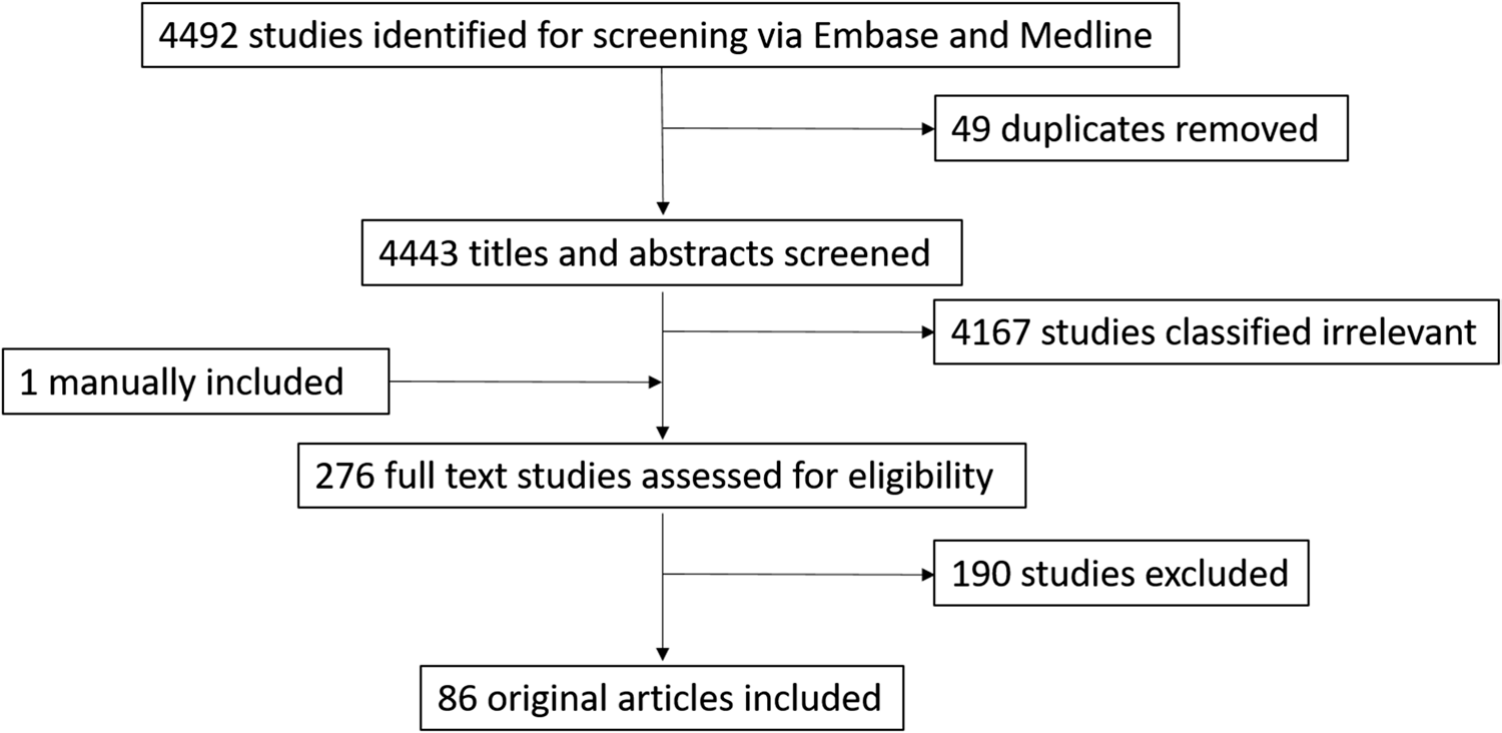
Flow diagram of systematic review search and screening results. The initial search strategy yielded 4492 references across two databases. Duplicate removal resulted in 4443 studies for title and abstract screening by two independent reviewers. 276 studies went forward to full text screening, and resulted in 86 studies for inclusion in this review

Of the initial 4492 studies identified, 49 were identified as duplicates. Following title and abstract screening of the remaining 4443 articles, 276 were selected for full text screening, and 1 was manually included (conference abstract identified in original literature search had further associated full text publication). Of these, 190 studies were excluded primarily because they did not meet the inclusion criteria, or contained characteristics of the exclusion criteria; including not meeting study design criteria (79), use of non-mesenchymal cells (27), no protective factors identified (35), extracellular vesicles or exosomes identified (3), or study was not of cardiovascular context (6). A number of studies were excluded for retraction (1), poor quality (2), duplication (3), conference abstracts (28), literature reviews (1), or inaccessible full text (5), and a further duplicate study was excluded manually following screening in Covidence. A final total of 86 original articles were included in this review (Fig. 1). The percentage of agreement on study inclusion was 87%, and the kappa score was 0.687; signifying substantial initial agreement.

### Study Characteristics & Quality Assessment

**Fig. 2:**
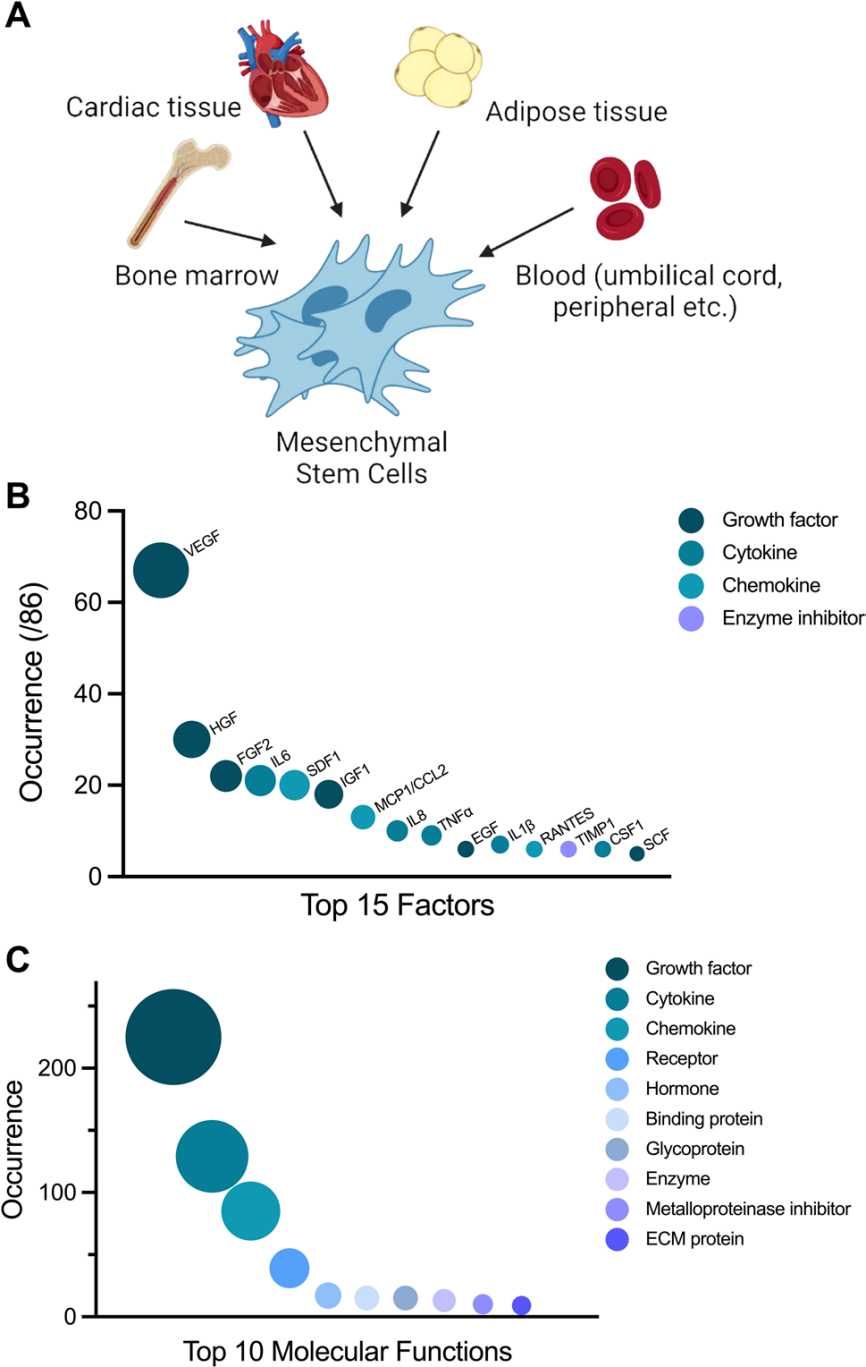
Commonly identified stem cell sources, their secreted paracrine factors, and associated molecular functions. (**a**) Primary sources stem cells of mesenchymal origin were derived from included bone marrow, cardiac tissue, adipose tissue, and blood. (**b**) The top 15 protective paracrine factors found to be secreted from cells of mesenchymal origin (**c**) The top 10 molecular functions of secreted factors*.* (**a**) Was created with BioRender.com

The stem cells used in these studies were primarily derived from bone marrow (59/86), cardiac tissue (16/86), and adipose tissue (11/86). Other sample sources included bone fragments (1/86), cortical bone (1/86), blood: umbilical cord blood (2/86), peripheral blood (1/86), menstrual blood (1/86), or healthy term placenta (1/86) (Fig. 2A). These samples were collected from human (31/86), rat (27/86), mouse (28/86), pig (1/86) or horse (1/86) subjects. A further study did not disclose the species the stem cells were derived from. Of the 86 articles included in this study based on identification of MSC paracrine factors, 35/86 further investigated the beneficial effects of stem cells in vitro. The functional effects of stem cell therapy were further assessed in 11/86 studies using ex vivo models of cardiac ischemia and in 44/86 using in vivo models of cardiac ischemia.

Within our quality assessment, we investigated the extent to which each of the included studies adhered to the International Society for Cellular Therapy (ISCT) proposed set of standards for identifying cells of mesenchymal origin [22] (Table 2). We found that only one of the studies met all recommended ISCT criteria in full. Adherence to plastic was reported by 55/86 studies, surface antigen expression was investigated by 62/86 studies, however these typically included a range of markers besides those recommended by the ISCT, and multipotency was reported by 38/86 studies. Only 11/86 studies scored higher than 80% in the quality assessment questionnaire. The results of the quality assessments for each article from both independent reviewers are detailed in supplementary information Table 3.

**Table 2:** Mesenchymal stem cell phenotyping and quality assessment findings of included studies

Author	Mean QA score (/17)	Cell type	Species	Source	Adherence	Multipotency	Positive markers	Negative markers	Reported or Cited?
[23]	9.5	APCs	Human	Adipose tissue	Yes	Not adipogenic	CD29, CD44, CD90, CD105, CD166	CD14, CD45, CD106	Reported
[24]	10	AD-MSCs	Human	Adipose tissue	–	Adipogenic, myogenic, osteogenic	CD73, CD105	CD34, CD45	Reported
[25]	10	AD-MSCs	Human	Adipose tissue	Yes	Adipogenic, myogenic, osteogenic	CD13, CD44, CD49b, CD90, CD105, HLA-Class I	CD15, CD34, CD133, c-kit, Flk-1, HLA-Class II	Cited
[26]	11.5	AD-MSCs	Mouse	Adipose tissue	–	–	CD29, CD44	CD45, CD73	Reported
[27]	10.5	AD-MSCs	Human	Adipose tissue	Yes	Adipogenic, chondrogenic, osteogenic	CD44, CD90, CD105	CD11b, CD14, CD34, CD45	Cited
[28]	13	AD-MSCs	Mouse	Adipose tissue	Yes	Adipogenic, osteogenic	CD90, CD105	CD31, CD45	Cited, reported
[29]	13.5	AD-MSCs	Human	Adipose tissue	–	Adipogenic, osteogenic	CD29, CD44, CD90	CD34, CD45	Reported
[21]	11.5	AD-MSCs, BM-MSCs	Human, rat	Adipose tissue, bone marrow	Yes	–	–	–	–
[30]	12	AD-MSCs, BM-MSCs	Rat	Adipose tissue, bone marrow	Yes	Adipogenic, osteogenic	–	–	Reported
[31]	8.5	AD-MSCs, CPCs	Mouse	Adipose tissue, cardiac tissue	–	–	Sca-1 (CPCs)	–	Reported
[32]	9	BF-MSCs	Human	Bone fragments	Yes	Adipogenic, chondrogenic, osteogenic	CD73, CD90, CD105	CD11b, CD19, CD34, CD45, HLA-DR	Reported
[33]	14	BM-MSCs	Mouse	Bone marrow	Yes	Adipogenic, osteogenic	CD44, Sca-1	CD45, CD90	Reported
[34]	8.5	BM-MSCs	Human	Bone marrow	–	–	–	–	–
[10]	15	BM-MSCs	Rat	Bone marrow	Yes	Adipogenic, chondrogenic, osteogenic	CD29, CD90	CD31, CD34, CD45	Reported
[35]	7.5	BM-MSCs	Rat	Bone marrow	–	–	–	–	–
[36]	12.5	BM-MSCs	Human	Bone marrow	Yes	–	–	–	Reported
[37]	11.5	BM-MSCs	Mouse	Bone marrow	–	Adipogenic, chondrogenic, osteogenic	CD105, Sca-1, SMA	CD14, CD45, c-kit	Reported
[38]	13	BM-MSCs	Rat	Bone marrow	–	–	–	–	–
[39]	10.5	BM-MSCs	Mouse	Bone marrow	Yes	–	–	–	Reported
[40]	14.5	BM-MSCs	Mouse	Bone marrow	Yes	–	–	–	Reported
[41]	16	BM-MSCs	Mouse	Bone marrow	Yes	–	–	–	Reported
[42]	14.5	BM-MSCs	Mouse	Bone marrow	Yes	–	–	–	Reported
[43]	15	BM-MSCs	Mouse	Bone marrow	Yes	–	–	–	Reported
[44]	13	BM-MSCs	Mouse	Bone marrow	Yes	–	–	–	Reported
[45]	10.5	BM-MSCs	Mouse	Bone marrow	Yes	Adipogenic, chondrogenic, osteogenic	CD29, CD44, CD105, Sca-1	CD11b, CD45	Reported
[46]	11	BM-MSCs	Human	Bone marrow	Yes	–	CD73, CD90, CD105	CD11b, CD14, CD34, CD45	Reported
[47]	10	BM-MSCs	Mouse	Bone marrow	Yes	Adipogenic, chondrogenic, osteogenic	CD44, CD90, CD105, Sca-1	CD14, CD34, CD45, c-kit	Cell surface markers reported; adherence & multipotency reported
[48]	11.5	BM-MSCs	Mouse	Bone marrow	Yes	Adipogenic, chondrogenic, osteogenic	CD34, CD106, Sca-1	CD11b, CD31, CD45, CD45R, CD90, c-kit, Flk-1, Ly-6C, Ly6G	Adhesion reported, Cell surface markers and multipotency cited
[49]	12	BM-MSCs	Rat	Bone marrow	Yes	–	CD29	CD11b	Reported
[50]	15.5	BM-MSCs	Rat	Bone marrow	Yes	–	CD29, CD44, CD90	CD14, CD34, CD45	Reported
[51]	10.5	BM-MSCs	Mouse	Bone marrow	–	Adipogenic, osteogenic	CD29, CD90, CD105	CD31, CD34, CD45, Flk-1	Reported
[52]	9.5	BM-MSCs	Mouse	Bone marrow	–	Adipogenic, osteogenic	CD29, CD90, CD105	CD34, CD45, c-kit	Reported
[53]	7.5	BM-MSCs	Rat	Bone marrow	–	–	CD44, CD90	CD34, CD45	Reported
[54]	8.5	BM-MSCs	Human	Bone marrow	Yes	Adipogenic, chondrogenic, osteogenic	CD29, CD44, CD105	CD34, CD45	Adhesion and multipotency cited cell surface markers reported
[55]	14	BM-MSCs	Rat	Bone marrow	Yes	–	CD44, CD90, CD105, Sca-1	CD34, CD45	Reported
[56]	10	BM-MSCs	Rat	Bone marrow	–	–	–	–	–
[57]	6.5	BM-MSCs	Rat	Bone marrow (commercial line)	–	–	–	–	–
[58]	13	BM-MSCs	Rat	Bone marrow	–	–	CD75, CD105, CD90	CD45	Reported
[59]	7.5	BM-MSCs	Mouse	Bone marrow	Yes	–	CD90, CD105, Sca-1	CD31, CD34, CD45	Reported
[60]	12	BM-MSCs	Rat	Bone marrow	Yes	–	–	–	Reported
[61]	7	BM-MSCs	Rat	Bone marrow	–	–	CD29, CD44, CD90	CD45	Reported
[62]	10	BM-MSCs	Mouse	Bone marrow	Yes	–	CD44	CD34, CD45, c-kit	Reported
[63]	9	BM-MSCs	Rat	Bone marrow	Yes	Adipogenic, osteogenic	–	–	Cited
[64]	9	BM-MSCs	Mouse	Bone marrow	Yes	Adipogenic, osteogenic	CD45, CD105, Sca-1	CD34, c-kit	Cited
[65]	11.5	BM-MSCs	Human	Bone marrow	–	–	–	–	Cited, inaccessible
[66]	11	BM-MSCs	Human	Bone marrow	Yes	Adipogenic, chondrogenic, osteogenic	CD73, CD90, CD105	CD11b, CD14, CD45	Reported
[67]	12	BM-MSCs	Human	Bone marrow	Yes	Adipogenic, osteogenic	CD29, CD90, CD105	CD31, CD34, CD45, CD133, c-kit	Cited
[68]	14.5	BM-MSCs	Mouse	Bone marrow	Yes	–	–	–	Cited
[13]	11	BM-MSCs	Human	Bone marrow	Yes	Adipogenic, chondrogenic, osteogenic	CD29, CD44, CD71, CD90, CD106, CD120a, CD124, SH2, SH3	CD14, CD34, CD45	Cited
[69]	11	BM-MSCs	Rat	Bone marrow	Yes	Adipogenic, chondrogenic, osteogenic	CD29, CD44, CD90, CD105	CD34, CD45	Multipotency cited adherence & cell surface markers reported
[70]	11	BM-MSCs	Human	Bone marrow	Yes	Adipogenic, chondrogenic, osteogenic	CD29, CD73, CD90, CD44, CD105, CD166	CD14, CD19, CD34, CD45, HLA-DR	Cited
[71]	7	BM-MSCs	Rat	Bone marrow	Yes	–	CD71, CD90, CD105, CD106, ICAM	CD14, CD34	Cited
[72]	13.5	BM-MSCs	Human	Bone marrow	Yes	Adipogenic, chondrogenic, osteogenic	CD44, CD73, CD90, CD166	CD34, CD45, HLA-DR	Cited
[73]	7.5	BM-MSCs	Human	Bone marrow	–	–	CD29, CD44, CD105, CD166	CD14, CD34, CD45	Reported
[74]	12.5	BM-MSCs	Rat	Bone marrow	Yes	Adipogenic, osteogenic	–	–	Cited
[75]	11	BM-MSCs	Human	Bone marrow	–	Adipogenic	CD49c, CD73, CD90, CD105	CD34, CD45, CD106, CD184	Reported
[76]	12.5	BM-MSCs	Rat	Bone marrow	Yes	–		CD45, CD90, CD44, CD29, CD34	Reported in methods, but no results available
[17]	10.5	BM-MSCs	Mouse	Bone marrow	Yes	–	CD34, c-kit, Flk-1	–	Cited
[77]	11.5	BM-MSCs	–	Bone marrow (commercial line)	–	–	–	–	–
[78]	8.5	BM-MSCs	Rat	Bone marrow	Yes	–	CD29, CD90, CD106	CD34	Reported
[79]	12	BM-MSCs	Rat	Bone marrow	Yes	–	CD29, CD71, CD90, CD106, c-kit	CD34, CD45	Cited
[80]	8.5	BM-MSCs	Mouse	Bone marrow	Yes	Adipogenic, chondrogenic, osteogenic	Stro-1	–	Reported
[81]	12	BM-MSCs	Rat	Bone marrow	Yes	Adipogenic, chondrogenic, osteogenic	–	CD34, CD45	Reported
[82]	12.5	BM-MSCs	Rat	Bone marrow	–	–	–	–	–
[83]	12.5	BM-MSCs	Human, rat	Bone marrow, cardiac tissue	–	Adipogenic, osteogenic	MSCs: CD90, CD105; CSC: c-kit	MSCs: CD34, CD45	Reported
[84]	9.5	BM-MSCs, AD-MSCs	Human, rat	Adipose tissue, bone marrow	Yes	–	AD-MSCs: CD90, CD105	AD-MSCs: CD45	Reported
[85]	8.5	BM-MSCs, En-MSCs	Human	Bone marrow (commercial line), menstrual blood	Yes	BM-MSCs (adipogenic, chondrogenic, osteogenic)	BM-MSCs: CD29, CD44, CD105; EnSCs: CD29, CD90, CD105, CD166	BM-MSCs: CD34, CD45; EnSCs: CD34, CD45, CD133	Adhesion and cell surface markers reported; Multipotency cited
[86]	11	CBSCs, CSCs	Mouse	Cardiac tissue, cortical bone	Yes	–	CD29, c-kit, Sca-1,	CD5, CD11b, CD34, CD45	Reported
[1]	11	CSCs	Human	Cardiac tissue	–	Endothelial, myogenic (cardiomyocyte, smooth muscle cell)	CD44, CD90, CD105	CD31, CD34, CD45	Reported
[87]	12	CSCs	Mouse	Cardiac tissue	–	–	PDGFRa, Sca1	Lin	Reported
[88]	13.5	CSCs	Pig	Cardiac tissue	–	–	CD29, CD44, CD90, CD105, SLA I	CD31, CD40, CD45, CD86 CD116, CD11R3, SLA II	Reported
[89]	13	CSCs	Mouse	Cardiac tissue	–	–	CD29, CD90, c-kit, Sca-1	CD31, CD34, CD45, Flk-1	Reported
[90]	11.5	CSCs	Human	Cardiac tissue	–	–	CD29, CD34, CD55, CD73	CD45, c-kit	Reported
[3]	9	CSCs	Mouse	Cardiac tissue	–	–	CD29, CD44, Sca-1	CD31, CD34, CD45, c-kit	Reported
[91]	7.5	CSCs	Human	Cardiac tissue	–	–	–	–	–
[92]	9.5	CSCs	Rat	Cardiac tissue	Yes	–	c-kit	–	Reported
[93]	15	CSCs	Human	Cardiac tissue	–	Myogenic (cardiomyocytes)	CD90, CD105	CD34	Reported
[94]	10	CSCs	Mouse	Cardiac tissue	Yes	–	Sca-1	–	Cited
[95]	9.5	CSCs	Mouse	Cardiac tissue	Yes	–	CD29, CD44, CD105	CD31, CD45, FLK1	Reported
[2]	13	CSCs	Rat	Cardiac tissue	Yes	–	c-kit (CSCs)	CD45 (CSCs)	Reported
[96]	9	CSCs	Human	Cardiac tissue	Yes	Adipogenic, chondrogenic, osteogenic	CD105	CD34	Reported
[97]	8	MSCs	Human	Unspecified (commercial line)	–	–	–	–	–
[98]	13	PB-MSCs	Horse	Peripheral blood	–	Adipogenic, chondrogenic, osteogenic	CD29, CD44, CD90, CD105	CD45, CD79	Reported
[15]	11.5	P-MSCs	Human	Placenta	Yes	Adipogenic, osteogenic	CD73, CD90, CD105, HLA-ABC	CD14, CD31, CD34, CD45, CD80, CD133, HLA-DR	Reported
[99]	14.5	UCB-MSCs	Human	Umbilical cord blood	Yes	Adipogenic, chondrogenic, osteogenic	CD29, CD44, CD73, CD90	CD14, CD34, CD45, CD133, CD144	Multipotency reported; adherence & cell surface markers cited
[100]	8.5	UCB-MSCs	Human	Umbilical cord blood	Yes	Adipogenic, chondrogenic, osteogenic	CD29, CD90, CD105	CD34, CD45, SSEA-3	Cited

Abbreviations: *AD-MSCs* adipose tissue derived mesenchymal stem cells, *APCs* adipose progenitor cells, *BM-MSCs* bone marrow derived mesenchymal stem cells, *CBSCs* cortical bone derived mesenchymal stem cells, *CD* cluster of differentiation, *c-kit* tyrosine-protein kinase Kit, *CPCs* cardiac progenitor cells, *En-MSCs* menstrual blood derived mesenchymal stem cells, *HLA* human leukocyte antigen, *MSCs* mesenchymal stem cells, *PB-MSCs* peripheral blood derived mesenchymal stem cells, *P-MSCs* placenta derived mesenchymal stem cells, *Sca-1* stem cell antigen 1, *SMA* smooth muscle actin, *UCB-MSCs* umbilical cord blood derived mesenchymal stem cells

**Table 3:** Identified paracrine factors and the effects of stem cell therapy in relevant in vitro cardiac models

Species	Cell type	Origin	Factors in conditioned media	In vitro model	Results	Author
Human	APCs	Adipose tissue	FGF2, IL6, TNFα, VEGF,	–	–	Bayes-Genis [23],
Human	AD-MSCs	Adipose tissue	ANGPT2, ANGII, FGF, FGF2, GCSF, GROα, HGF, IFNγ, IGF1, IL1, IL1α, IL1β, IL2, IL4, IL5, IL6, IL8, IL10, IL12, IL13, IL15, IL17, IL23, MCP1, MCP3, MMP1, MMP2, MMP3, PDGF, PDGFBB, SCF, SDF1, TGFβ, TIMP1, TIMP2, TNFα, VEGF	hDMECs [27] or NRCs [27, 29]; AD-MSC co-culture or conditioned media; Hypoxia [27, 29]	↓ apoptosis [27, 29]; ↑ tube formation [27]	Adutler-Lieber [24], Anderson [21] *, Figeac [25], Li [84], Sadat [27], Yang [29],
Human	BF-MSCs	Bone fragments	BDNF, EGF, FGF2, HGF, NGF, NT3, NT4, SDF1α, VEGFα, IL1β, IL6, IL8	–	–	Montzka [32]
Human	BM-MSCs	Bone marrow	AgRP, Angiogenin, ANGPT 1, ANGPT2, Amphiregulin, Axl, BDNF, BLC, BMP4, BMP6, βNGF, BTC, CCL28, CK β 8–1, CNTF, CTACK, DKK1, Dtk, EGF, EGFR, EMMPRIN, ENA78, endoglin, Eotaxin, Eotaxin 2, Eotaxin 3, Fas, FGF2, FGF4, FGF6, FGF7, FGF9, Flt3 Ligand, Fractalkine, GCP2, GCSF, GDF15, GDNF, GITR Ligand, GITR, GMCSF, GRO, GROα, HCC4, HGF, I309, ICAM1, ICAM3, IFNγ, IGF1, IGFBP1, IGFBP2, IGFBP3, IGFBP4, IGFBP6, IGF-I SR, IL1α, ILIβ, IL1Rα, IL1R4/ ST2, IL1RI, IL2, IL2Rα, IL3, IL4, IL5, IL6, IL6R, IL7, IL8, IL10, IL11, IL12 p40, IL12 p70, IL13, IL15, IL16, IL17, ITAC, LEP, LIGHT, MCP1, MCP2, MCP3, MCP4, MCSF, MDC, MIF, MIG, MIP1α, MIP1β, MIP1δ, MIP3α, MIP3β, MSPα, NAP2, NT3, NT4, Osteoprotegerin, Oncostatin M, OPN, PAI-1, PARC, PDGF, PDGFAA, PDGFBB, PECAM1, PIGF, PTX3, RANTES, SCF, SDF1, SDF1α, Sgp130, sTNF RII, sTNF RI, TARC, TECK, TGFβ1, TGFβ3, TIMP1, TIMP2, TPO, TNFα, TNFβ, TRAIL R3, TRAIL R4, Tsp1 uPAR, VCAM1, VEGF, VEGFD, XCL1, YKL40	CMs [36], HUVECs [13, 46, 54, 72] or NRCs [13, 70]; BM-MSCs co-culture or conditioned media; Hypoxia [13, 70]	↓ apoptosis[13, 70]; ↑ proliferation [13, 36, 72], tube formation [13, 46, 72], cell aggregation structures [54], migration [72]	Alrefai [34], Baffour [36], Deng [46], Jiang [85], Li [54], Li [84], Paquet [65, 66], RanjendranNair [67], See [13], Song [70], Tang [83], Thej [72], Wairiuko [73], Windmolders [75]
Human	CSCs	Cardiac tissue	Angiogenin, ANGPT1, ANGI, ANGII, CD26, ET1, FGF2 GMCSF, GRO molecules, HGF, IGF1, IGFBP1, IGFBP2, IGFBP3, IL6, IL8, MCP1, miR132, OPG, SCF, SDF1, SDF1a, uPA, VEGF	CMs [1, 93], HMEC-1 s [90], or HUVECs [91, 93]; conditioned media; H/R [1, 93]	↓ apoptosis [1, 93]; ↑ EC proliferation, migration, and tube formation [90, 91, 93]	Avolio [1], Czapla [96], Fanton [90], Latham [91], McQuaig [93]
Human	En-MSCs	Menstrual blood	VEGF, TGFB2, EGF	HUVECs or NRCs; En-MSC co-culture or conditioned media; hypoxia	↓ apoptosis; ↑ proliferation, tube formation, tube length	Jiang [85]
Human	MSCs	Unspecified (commercial line)	VEGF, HGF, IL6, PLGF, Adrenomedullin	CMs (HL-1) or HUVECs; conditioned media; hypoxia	↓ apoptosis	Iso [97]
Human	P-MSCs	Placenta	Angiogenin, EGF, ENA78, FGF2, GRO, IFNɣ, IGF1, IL6, IL8, LEP, MCP1, PDGFBB, PIGF, RANTES, TGFβ1, TIMP1, TIMP2, TPO, VEGF, VEGFD	CMs (H9c2) or EPCs; conditioned media; H/R	↓ apoptosis; ↑ tube formation	Danieli [15]
Human	UCB-MSCs	Umbilical cord blood	ANGPT2, EGF, FGF2, HGF, IL6, VEGF	CMs (HL-1); conditioned media; hypoxia	↓ apoptosis	Bader [99, 100]
Rat	AD-MSCs	Adipose tissue	Adrenomedullin, ANGPT2, FGF2, HGF, IGF1, IL6, LEP, PAI1, SDF1, SDF1α, TNFα, VEGF	–	–	Anderson [21], Li [84], Nakanishi [30]
Rat	BM-MSCs	Bone marrow	Activin A, Adrenomedullin, ANGII, Anxa1, bNGF, CINC1 Decorin, FGF2, Flt3 ligand, FSTL1, Gas6, HGF, Hsp90b1, IGF, IGF1, IL1β, IL6, IL10, IL13, LEP, LOC286987, MIF, NRP2, Nme2, PAI1, PDGFAA, Scg3, SCF, SDF, SDF1, SDF1α, Tagln, TGFβ, TNFα, Tpm, Tpm1, VEGF	CMs [38, 79], CMs (H9c2) [57, 58], NRCs [10, 56, 69, 78]; BM-MSC co-culture or conditioned media; hypoxia [38, 56, 78, 79] or H/R [10, 57, 58]	↓ apoptosis [38, 56–58, 58, 69, 78], LDH activity [10]; ↑ viability [10], cell-cycle re-entry [78], proliferation [56, 58]	Anderson [21], Angoulvant [10], Augustin [35], Cai [38], Fan [49], Fan [50], Ju [53], Li [84], Li [55], Li [56], Li [57], Lin [58], Luo [60], Mao [61], Meng [63], Nakanishi [30], Shan [69], Song [71], Wang [74], Xia [76], Yu [78], Zeng [79], Zhang [81, 82]
Rat	CSCs	Cardiac tissue	ANGPTL2, IGF1, VEGF	–	–	Bao [2], Li [92]
Mouse	BM-MSCs	Bone marrow	Ang1, ANGPTL3, CCL22, CX3CL1, Cystatin C, CD40, EPO, FGF2,Gas6, GROα, HGF, HIF1α, ICAM1, IFNγ, IGF, IGF1, IL6, IL10, IL12, IL15, IL18, IL28A/B, KGF, LEP, LIF, MCP1, MCSF, MIG, MIP1α, MIP1β, MIP2, MMP2, MMP9, OPN, PDGFBB, PTX3, POSTN, PGE2, PLGF, Proliferin, PCSK9, RANTES, SDF1, SDF1a, PAI1, TGFβ, TNFα, VEGF, VEGF1	CFbs [39], CMs [47], CMs (H9c2) [37], HUVECs [51] or NRCs [44, 68]; BM-MSCs co-culture or conditioned media; hypoxia [37, 39, 47] or H/R [44]	↓ fibroblast activation, collagen [39], mitochondrial membrane potential [37], apoptosis [37, 44, 47], LDH activity [44, 47]; ↑ proliferation [44, 68], tube formation [51]	Abarbanell [33], Burlacu [37], Chen [39] *, Crisostomo [40–43], Dai [44], Daltro [45], Deuse [47], Erwin [48], Huang [52], Huang [51], Lu [59], Markel [62], Page [64], Sassoli [68], Xu [17], Zhang [80]
Mouse	AD-MSCs	Adipose tissue	4-1BB, ACE, Amphiregulin, Axl, bFGF CD27, CD36, CD40 ligand, CTF1, CXCL16, DCN, DKK1, E-cadherin, EGF, Epiregulin, GCSF, GITR ligand, GZMB, GAS1, HAI-1, HGF, IGFBP6, IL1ra/IL1F3, IL6R, IL17B, IL17F, IL20, IL21, Il28, JAMA, LGALS1, MAdCAM1, MCP1, MCSF, MFGE8, MIP1ɣ, MIP3α, MMP10, MMP13, MME, OPN, PTX3, Prolactin, RAGE, RANTES, SDF1a, sTNFRI, sTNFRII, TACI, TWEAK R, VCAM1, VEGF, VEGFR1	RCAECs; conditioned media [28]; HUVECs, conditioned media [26]	↑ tube formation [28], wound closure [26]	Liu [26, 31], Yan [28]
Mouse	CSCs	Cardiac tissue	ANGPT2, ANGI, B2MG, CCL7, COL12, CSF1, CTGF, DAG1, DTK, ENG, EPGN, FGF2, GAS6, GCSF, GDF6, GDF8, GRN, HGF, IGF1, IGFBP2, IL1AP, IL11, IL15Ra, IL17E, INHBA, LG3BP, LRP1, MCSF, MIF, MIME, MRC2, MYDGF, NENF, OPN, PDGF, Pro-MMP9, SCF, SDF, SDF1, SFRP1, TGFβ2, TIMP1, TRAIL, VCAM1, VEGF, VEGFD, WISP2,	–	–	Constantinou [87], Cui [89], Duran [86] *,, Huang [3], Samal [94], Zhao [95]
Mouse	CBSCs	Cortical bone	ANG1, FGF2, HGF, IGF1, PDGF, SCF, SDF1, VEGF	–	–	Duran [86] *
Mouse	CPCs	Cardiac tissue	4-1BB, ACE, Amphiregulin, Axl, CD27, CD36, CD40 ligand, CTF1 CXCL16, DCN, DKK1, E-cadherin, ENG, EGF, EPGN, Epiregulin, GCSF, GITR ligand, GZMB, Growth arrest specific 1, HAI-1, HGF, IGFBP6, IL1R4/ST2L, IL1RA/IL1F3, IL6R, IL11, IL17B, IL17E, IL17F, IL20, IL21, IL28, JAMA, LGALS1, MAdCAM1, MCP1, MCSF, MFGE8, MIP1γ, MIP3α, MME, OPN, PTX3, Prolactin, Pro-MMP9, RAGE, RANTES, sTNF RI, sTNF RII, TACI, TIMP1, TWEAK, TWEAK R, VCAM1, VEGF, VEGF R1	–	–	Liu [31]
Pig	CSCs	Cardiac tissue	CCL2, CXCL12, HGF, IGF1, TGFB1	–	–	Crisostomo [88]
Horse	PB-MSCs	Peripheral blood	activin A, ANGPT1, ET1, IGFBP2, IL8, PDGFAA, uPA, VEGF	ECs; conditioned media	↑ proliferation, tube formation,	Bussche [98]

The “Model” and “Results” column refers to the specifics of the identified studies where further investigations were undertaken in vitro. Some studies only profiled the secreted paracrine factors of cells, thus the “Author” column references all studies through which factors were identified within a given row

Abbreviations: *4-1BB* tumour necrosis factor receptor superfamily member 9, *ACE* angiotensin converting enzyme, *AD-MSC* adipose tissue derived mesenchymal stem cells, *AgRP* Agouti-related protein, *ANG* angiotensin, *ANGPT* angiopoietin, *ANGPTL* angiopoietin like, *Anxa1* annexin A1, *Axl* AXL receptor tyrosine kinase, *B2MG* beta-2-microglobulin, *BDNF* brain derived neurotrophic factor, *BLC* beta lymphocyte chemoattractant, *BM-MSCs* bone marrow derived mesenchymal stem cells, *BMP* bone morphogenetic protein, *BTC* probetacellulin, *CCL* C–C motif chemokine, *CD* cluster of differentiation, *CFbs* cardiac fibroblasts, *CK β 8–1* C–C motif chemokine 23, *CMs* cardiomyocytes, *CNTF* ciliary neurotrophic factor, *COL* collagen, *CSF1* macrophage colony stimulating factor 1, *CTACK* C–C motif chemokine 27, *CTF* cardiotrophin, *CTGF* cellular communication network family member 2, *CX3CL1* fractalkine, *CXCL* C-X-C motif chemokine, *DAG1* dystroglycan, *DCN* decorin, *DKK1* Dickkopf related protein 1, *EC* endothelial cell, *EGF* epidermal growth factor, *EGFR* epidermal growth factor receptor, *ENA78* C-X-C motif chemokine 5, *EMMPRIN* Basigin, *ENG* endoglin, *En-MSCs* menstrual blood derived mesenchymal stem cells, *EPCs* endothelial progenitor cells, *EPGN* epigen, *EPO* erythropoietin, *ET* endothelin, *Fas* tumour necrosis factor receptor superfamily member 6, *FGF* fibroblast growth factor, *Flt3* Receptor-type tyrosine-protein kinase, *FSTL1* follistatin-related protein 1, *GAS* growth arrest specific protein, *GCP2* C-X-C motif chemokine 6, *GCSF* granulocyte colony-stimulating factor, *GDF* growth/differentiation factor, *GDNF* glial cell line derived growth factor, *GITR* tumour necrosis factor receptor superfamily member 18, *GMCSF* granulocyte–macrophage colony-stimulating factor, *GRO* growth regulated, *GRN* progranulin, *GZMB* granzyme B, *HAI-1* Kunitz-type protease inhibitor 1, *HGF* hepatocyte growth factor, *hDMECs* human dermal microvascular endothelial cells, *hMEC-1* human microvascular endothelial cells, *HIF* hypoxia inducible factor, *I309* C–C motif chemokine 1, *H/R* hypoxia/ reperfusion, *Hsp90b1* endoplasmin, *HUVECs* human umbilical vein endothelial cells, *ICAM* intercellular adhesion molecule 1, *IFN* interferon, *IGF* insulin like growth factor, *IGFBP* insulin like growth factor binding protein, *IL* interleukin, *INHBA* inhibin beta A chain, *ITAC* C-X-C motif chemokine 11, *KGF* fibroblast growth factor 7, *LDH* lactate dehydrogenase, *LEP* leptin, *LGALS1* galectin 1, *LG3BP* galectin-3 binding protein, *LIF* leukemia inhibitory factor, *LOC286987* hemiferrin, *LRP1* prolow-density lipoprotein receptor-related protein 1, *MAdCAM* mucosal addressin cell adhesion molecule, *MCP* monocyte chemoattractant, *MCSF* macrophage colony-stimulating factor 1, *MDC* C–C motif chemokine 22, *MIF* macrophage migration inhibitory factor, *MIG* macrophage induced gene, *MIP* macrophage inflammatory protein, *miR* microRNA, *MFGE8* lactadherin, *MIME* mimecan, *MME* neprilysin, *MMP* matrix metalloproteinase, *MRC2* c-type mannose receptor 2, *MYDGF* myeloid-derived growth factor, *NAP* neutrophil activating peptide, *NENF* neudesin, *NGF* beta nerve growth factor, *NRCs* neonatal rat cardiomyocytes, *NRP2* neuropilin 2, *NT* neurotrophin, *OPG* osteoprotegerin, *OPN* osteopontin, *PAI1* plasminogen activator inhibitor 1, *PARC* C–C motif chemokine 18, *PCSK9* Proprotein convertase 9, *PDGF* platelet derived growth factor, *PECAM1* platelet endothelial cell adhesion molecule, *PGE2* prostaglandin E2, *PIGF* phosphatidylinositol-glycan biosynthesis class F protein, *PLGF* placenta growth factor, *POSTN* periostin, *PTX* pentraxin, *RAGE* receptor for advanced glycosylation end products, *RANTES* C–C motif chemokine 5, *SCF* stem cell factor, *Scg3* secretogranin 3, *SDF* stromal cell derived factor, *SFRP* secreted frizzled-related protein, *Sgp130* interleukin 6 receptor subunit beta, *sTNF R* soluble tumour necrosis factor receptor, *TACI* tumour necrosis factor receptor superfamily 13B, *Tagln* transgelin, *TARC* C–C motif chemokine 17, *TECK* C–C motif chemokine 25, *TGFβ* transforming growth factor beta, *TIMP* metalloproteinase inhibitor, TNFα, tumour necrosis factor alpha, *TNFβ* tumour necrosis factor beta, *Tpm* tropomyosin, *TPO* thrombopoietin, *Tsp1* thrombospondin 1, *TRAIL R3* tumour necrosis factor receptor superfamily member 10c, *TRAIL R4* tumour necrosis factor receptor superfamily member 10c, *TWEAK* tumour necrosis factor ligand superfamily member 12, *TWEAKR* tumour necrosis factor receptor superfamily member 12A, *uPA* urokinase plasminogen activator, *uPAR* urokinase plasminogen activator surface receptor, *VCAM* vascular cell adhesion protein 1, *VEGF* vascular endothelial growth factor, *VEGFR* vascular endothelial growth factor receptor, *WISP2* cellular communication network family member 5, *XCL1* lymphotactin, *YKL40* Chitinase-3-like protein 1, * all factors identified in cell lysate

### In Vitro – Commonly Identified Factors and their Effects

Across the 86 included articles, a total of 234 different factors were identified using a range of techniques including ELISA, qPCR, western blot, immunostaining, mass spectrometry, immunoassays, and microarrays.

The most commonly identified factors (Fig. 2B) directly released by MSCs were VEGF (67/86), hepatocyte growth factor (HGF, 30/86), fibroblast growth factor 2 (FGF2, 22/86), interleukin-6 (IL6, 21/86), stromal cell-derived factor 1 (SDF1, 20/86), insulin like growth factor 1 (IGF1, 18/86), C–C motif chemokine 2 (MCP1/CCL2, 13/86), interleukin-8 (IL8, 10/86), tumour necrosis factor alpha (TNFα, 9/86), interleukin-1β (IL1β, 7/86), C–C motif chemokine 5 (CCL5, 6/86), epidermal growth factor (EGF, 6/86), metalloproteinase inhibitor 1 (TIMP1, 6/86), macrophage colony-stimulating factor 1 (CSF1, 6/86), and stem cell factor (SCF, 5/86). When categorized by molecular function (Fig. 2C), the identified factors were commonly classified as growth factors, cytokines, chemokines, receptors, and hormones.

The beneficial effects of factors released by stem cells in vitro were investigated in 38/86 studies by utilizing primary adult cardiomyocytes (CMs) (5/38), primary neonatal rat cardiomyocytes (NRCs) (12/38), CM cell lines (HL-1, H9c2, AC16) (8/38), or endothelial cell lines (hDMECs, HUVECs, HMEC-1) (17/38). These cells were co-cultured with stem cells or their conditioned media under normoxic or hypoxic conditions, and the effects on angiogenesis, apoptosis, and proliferation studied when compared to appropriate controls (e.g. untreated, vehicle treated, or non-reparative cell type treated groups). The articles included in this study demonstrated that factors released by stem cells of mesenchymal origin (including human AD-MSCs, BM-MSCs, CSCs, En-MSCs, P-MSCs, and UCB-MSCs, as well as rat and mouse BM-MSCs) can reduce CM and endothelial cell apoptosis under hypoxic conditions, promote tube formation in endothelial cells, and increase endothelial cell proliferation or migration as further detailed in Table 3.

### Ex Vivo and In Vivo Cardiac Models—Functional Associations of Stem Cell Therapy

Of the articles included in this study, 11/86 performed ex vivo experiments largely comprising of Langendorff experimental models of ischemia/ reperfusion (I/R) injury; and 45/86 performed in vivo experiments in which MI was induced using permanent or transient ligation of the left anterior descending (LAD) artery.

For ex vivo experiments, BM-MSCs, CSCs, or their conditioned media were perfused pre- or post-I/R injury, and resulted in overall improvements in cardiac function including increased left ventricular developed pressure (LVDP), right ventricular developed pressure (RVDP), contractility, and compliance, and reduced end diastolic pressure (EDP) during Langendorff perfusion, when compared to appropriate controls (e.g. untreated, vehicle treated, or non-reparative cell type treated groups).

For in vivo experiments, AD-MSCs, APCs, BM-MSCs, CBSCs, CPCs, CSCs, En-MSCs, and P-MSCs derived from human, rat, or mouse were utilized as whole cell or conditioned media therapy. The broad range of stem cells of mesenchymal origin studied in the included articles resulted in a range of functional improvements as measured by echocardiography or haemodynamics when compared to appropriate controls (e.g. untreated, vehicle treated, or non-reparative cell type treated groups). Treated hearts had decreased infarct size, reduced signs of cardiac remodelling, improvements in systolic and diastolic function, and reduced fibrosis. Other signs of improvement in cardiac function reported included increased vascular density and reduced CM apoptosis. Specific results of both ex vivo and in vivo experiments are expanded upon in Table 4.

**Table 4:** Functional associations of stem cell therapy in ex vivo and in vivo cardiac models

Species	Cell Type	Factors Identified	Model	Results	Author
Ex vivo					
Human	BM-MSCs	PDGFAA	Atrial appendage tissue was cultured in presence of BM-MSC conditioned media	BM-MSC conditioned media ↑ cell migration from tissue; cells had CSC phenotype	Windmolders [75]
Human	BM-MSCs	HGF, VEGF	Rat Langendorff; I/R; BM-MSC treatment pre- I/R	BM-MSC treated ↑ RVDP, + dP/dT (contractility), -dP/dT (compliance) at end reperfusion	Wairiuko [73]
Rat	BM-MSCs	IGF1, VEGF	Rat Langendorff; cold I/R; BM-MSC conditioned media infusion pre-reperfusion	BM-MSC conditioned media ↓ creatine kinase, infarct size	Angoulvant [10]
Rat	BM-MSCs	VEGF	Rat Langendorff; I/R; BM-MSC treatment pre-I/R	BM-MSC treated: ↑ LVDP, + dP/dT (contractility), -dP/dT (compliance) at end reperfusion	Luo [60]
Mouse	BM-MSCs	IL-10, TNFα, VEGF	Rat Langendorff; I/R; BM-MSC treatment pre- [33, 40, 41, 43, 48, 62] or post-I/R [40, 41, 43]	BM-MSC treated: ↑ LVDP, + dP/dT (contractility), -dP/dT (compliance), myocardial VEGF; ↓ EDP at end reperfusion	Abarbanell [33], Crisostomo [40, 41, 43], Erwin [48], Markel [62]
Mouse	BM-MSCs, CSCs	HGF, IGF-1 SDF-1, VEGF	Mouse Langendorff; I/R; CSC, BM-MSC, CSC conditioned media treatment before I/R	BM-MSCs, CSCs, and CSC conditioned media: ↑ LVDP, + dP/dT (contractility), -dP/dT (compliance) at end reperfusion	Huang [3]
In vivo					
Human	APCs, BM-MSCs	FGF2, IL6, TNFα, VEGF	Mouse and rat MI by LAD ligation; IM injection APCs or BM-MSC post-MI	APCs ↓ infarct size; ↑ LVEF, FS, LV wall thickness; BM-MSCs had no effect	Bayes-Genis [23]
Human	AD-MSCs	FGF2, HGF, IL1, TGFβ, VEGF	Rat MI by LAD ligation; IM injection of normoxic or hypoxic ASC conditioned media 30 min post-MI	Hypoxic ASC conditioned media ↓ infarct size; ↑ LVEF, LVSP, + dP/dT (contractility), -dP/dT (compliance);	Yang [29]
Human	AD-MSCs	FGF2, GCSF, GROα, HGF, IL6, MCP1, MCP3,, MMP1, MMP2, MMP3, PDGFBB, SCF, SDF1, TIMP1, TIMP2, VEGF	Mouse LAD I/R; IM injection ASCs post-MI	ASCs ↓ infarct size; ↑ LVEF, vessel density	Figeac [25]
Human, rat	AD-MSCs, BM-MSCs	CXCL12 (SDF1), HGF	Rat LAD I/R; collagen micro-sponges with CXCL12 (SDF1) or HGF placed on infarcted anterior wall	HGF: ↓ infarct size, LVEDD, CM apoptosis at BZ; ↑ LVEF, myocardial HGF; CXCL12 (SDF1) sponges had no effect	Anderson [21]
Human, rat	AD-MSCs, BM-MSCs	ANGPT2, FGF2, HGF, IGF1, SDF1, VEGF	Mouse MI by LAD ligation; IM injection of BM-MSCs or AD-MSCs post-MI	AD-MSCs and BM-MSCs ↓ infarct perimeter, CM apoptosis; ↑ wall thickness,	Li [84]
Human	BM-MSCs	AgRP, Amphiregulin, Angiogenin, ANGPT2, Axl, BDNF, BLC, BMP4, BMP6, bNGF, BTC, CCL28, CK β 8–1, CNTF, CTACK, Dtk, EGF, EGFR, ENA78, Eotaxin, Eotaxin 2, Eotaxin 3, Fas, FGF2, FGF4, FGF6, FGF7, FGF9, Flt3 Ligand, Fractalkine, GCP2, GCSF, GDNF, GITR Ligand, GITR, GMCSF, GRO, GROα, HCC4, HGF, I309, ICAM1, ICAM3, IFNγ, IGF1, IGFBP1, IGFBP2, IGFBP3, IGFBP4, IGFBP6, IGF-I SR, IL1α, ILIβ, IL1rα, IL1R4/ ST2, IL1RI, IL2, IL2Rα, IL3, IL4, IL5, IL6, IL6R, IL7, IL8, IL10, IL11, IL12 p40, IL12 p70, IL13, IL15, IL16, IL17, ITAC, LEP, LIGHT, XCL1, MCP1, MCP2, MCP3, MCP4, MCSF, MDC, MIF, MIG, MIP1α, MIP1β, MIP1δ, MIP3α, MIP3β, MSPα, NAP2, NT3, NT4, Osteoprotegerin, Oncostatin M, PARC, PDGF, PDGFBB, PIGF, RANTES, SCF, SDF1, Sgp130, sTNF RII, sTNF RI, TARC, TECK, TGFβ1, TGFβ3, TIMP1, TIMP2, TPO, TNFα, TNFβ, TRAIL R3, TRAIL R4, uPAR, VEGF, VEGFD	Rat MI by LAD ligation; IM injection of BM-MSCs [13, 46, 70, 83] or BM-MSC conditioned media [13, 34] directly [46], 15 m [34], 48 h [13], 7d [83] or 10d [70] post-MI	BM-MSCs and BM-MSC conditioned media ↓ infarct size, LVEDP, LVEDD, LVESD, CM apoptosis, fibrosis; ↑ LVEF, FS, LVSP, + dP/dT (contractility), -dP/dT (compliance), vessel density; no BM-MSC engraftment	Alrefai [34], Deng [46], See [13], Song [70], Tang [83]
Human	BM-MSCs	Angiogenin, Dkk1, EMMPRIN, Endoglin, GDF15, IGFBP3, IL6, IL8, MCP1, MCP3, MIF, OPN, PAI-1, PDGFAA, PECAM1, PTX3, SDF1a, Tsp1, uPAR, VCAM1, VEGF, YKL40	Mouse MI by LAD ligation; IM injection of BM-MSCs post-MI	BM-MSCs had no effect	[66]
Human	CSCs	Angiogenin, ANGPT1, ANGI, ANGII, FGF2, GRO molecules, HGF, IL6, IL8, miR132, OPG, SCF, SDF1, SDF1a, VEGF	Mouse MI by LAD ligation; IM injection of CSCs directly [1] or 7d [91, 96] post-MI	CSCs ↓ infarct size, CM apoptosis, CM hypertrophy at BZ and RZ, fibrosis; ↑ LVEF, vessel density in BZ and IZ, CM proliferation; low CSC engraftment and differentiation	Avolio [1], Czapla [96], Latham [91]
Human	En-MSCs	EGF, TGFB2, VEGF	Rats MI by LAD ligation; IM injection of EnSCs 30 m post-MI	En-MSCs ↓ infarct size, CM apoptosis; ↑ LVEF, FS, vessel density, cell proliferation	Jiang [85]
Human	MSCs	Adrenomedullin, HGF, IL6, PLGF, VEGF	Mouse MI by LAD ligation; IV injection of MSCs at 1, 8, and 15d post-MI	MSCs ↓ LVSD, fibrosis; ↑ FS; no MSC engraftment	Iso [97]
Human	P-MSCs	Angiogenin, EGF, ENA78, FGF2, GRO, IFNɣ, IGF1, IL6, IL8, LEP, MCP1, PDGFBB, PIGF, RANTES, TGFβ1, TIMP1, TIMP2, TPO, VEGF, VEGFD	Rat LAD I/R; IM injection of P-MSC conditioned media 10 m post-ischemia	P-MSCs conditioned media ↓ necrotic area, CM apoptosis in BZ and IZ; ↑ LV wall thickness, vessel density at BZ;	Danieli [15]
Human	UCB-MSCs	VEGF	Rat MI by LAD ligation; IM injection of UCB-MSC conditioned media post-MI	UCB-MSC conditioned media ↓ fibrosis	[100]
Rat	BM-MSCs	FGF2, Gas6, HGF, IGF1, IL1β, IL10, SDF, SDF1, TGFβ, TNFα, VEGF	Rats MI by LAD ligation; BM-MSC sheet treatment [35], IV [58, 81], or IM injection of BM-MSC conditioned media [79], or BM-MSCs [58] directly [53, 69], 30 m [38], 60 m [50, 63], 1d [81], 7d [58, 74] post-MI	BM-MSCs, BM-MSC conditioned media, and BM-MSC sheets ↓ infarct size, LVEDD, LVESD, LVEDP, CM apoptosis, fibrosis; ↑ LVEF, FS, LV wall thickness, LVSP, + dP/dT (contractility), -dP/dT (compliance), vessel density in BZ and IZ; BM-MSCs detected in myocardium at 3d and 4wk post-MI	Augustin [35], Cai [38], Fan, Ju [53] [50], Lin [58], Meng [63], Shan [69], Wang [74], Zeng [79], Zhang [81]
Rat	BM-MSCs	HGF, IL6, IGF1, SCF, SDF1, VEGF	SD rats; LAD I/R; IM injection of BM-MSCs 2 h [61] or 7d [55] post-reperfusion	BM-MSCs ↓ LVESD, LVEDV, LVESV, infarct size, CM apoptosis; ↑ LVEF, FS, angiogenesis	Li [55], Mao [61]
Rat	CSCs	ANGPTL2, IGF1, VEGF	Rat MI by LAD ligation; IM injection of CSCs directly [92] and 28d [2] post-MI	CSCs ↓ infarct size, CM apoptosis, fibrosis; ↑ LVEF, FS, vessel density; no engraftment or differentiation	Li [92], Bao [2]
Mouse	AD-MSCs	bFGF, HGF, MMP10, MMP13, SDF1a, VEGF	C57BL/6 J mice; MI by LAD ligation and LAD I/R; IM injection of AD-MSCs directly post MI	AD-MSCs ↑ LVEF, LVEDV, ↓ LVESV	Yan [26, 28]
Mouse	BM-MSCs	FGF2, LEP, PLGF, VEGF,	Mouse MI by LAD ligation; IV [80] or IM injection of normoxic or hypoxic BM-MSCs directly [39, 80] or 1wk [52] post-MI	BM-MSCs ↓ infarct size, LVDD, fibrosis, myofibroblasts; ↑ LVEF, FS, vessel density, angiogenesis, blood flow, tubulogenesis; BM-MSC engraftment in IZ and BZ 1wk post-injection; no differentiation	Chen [39], Huang [52], Zhang [80]
Mouse	BM-MSCs	HGF, VEGF	Mouse MI by LAD ligation; IM injection of BM-MSCs with or without VEGF or HGF post-MI	BM-MSCs injected with HGF or VGEF ↓ infarct size; ↑ LVEF; no engraftment 7d post-injection	Deuse [47]
Mouse	BM-MSCs	FGF2, IGF1, SDF1, VEGF	C57BL/6 mice; irradiated & GFP + bone marrow transplant; MI by LAD ligation; IP injection of SCF 4 h post MI to 6d post-MI	SCF treatment ↓ LVIDD, LVISD; ↑ contractility, ventricular end-systolic elastance, GFP^+^ BM-MSC mobilisation;	Xu [17]
Mouse	CBSCs, CSCs	ANG1, DTK, GDF8, FGF2, HGF, IGF1, IGFBP2, IL15Ra, MCSF, OPN, PDGF, SCF, SDF1, TRAIL, VEGF	Mouse MI by LAD ligation; IM injection of CBSCs [86], CSCs [86, 95], or CSC conditioned media [87] post-MI	CBSCs and CSCs ↑ LVEF, FS, LV wall thickness, vessel density; CSCs ↓ CM apoptosis; CBSCs differentiate into functional cardiomyocytes, vascular smooth muscle cells, and endothelial cells	Constantinou [87], Duran [86], Zhao [95]
Mouse	CPCs	4-1BB, ACE, Amphiregulin, Axl, CD27, CD36, CD40 ligand, CTF1, CXCL16, DCN, DKK1, E-cadherin, EGF, ENG, EPGN, Epiregulin, GAS1, GCSF, GITR ligand, GZMB, HAI1, HGF, IGFBP6, IL17B, IL17E, IL17F, IL1RA/IL1F3, IL1R4/ST2L, IL11, IL20, IL21, IL28, IL6 R, JAMA, LGALS1, MAdCAM1, MCSF, MCP1, MFGE8, MIP1γ, MIP3α, MME, OPN, PTX3, Prolactin, Pro-MMP9, RAGE, RANTES, sTNF RI, TACI, TIMP1, TWEAK, TWEAK R, VCAM1, VEGF, VEGF R1	C57BL/6 J; MI by LAD ligation; IM injection of CPCs with nanopeptides post-MI	CPCs ↓ infarct size, LVIDD, LVISD; ↑ FS	Liu [31]
Pig	CSCs	CCL2, CXCL12, HGF, IGF1, TGFB1	Large white pigs; LAD I/R; IC injection of CSCs 7d post MI	CSCs ↓ infarct size; ↑ LVEF	Crisostomo [88]

Abbreviations: *4-1BB* tumour necrosis factor receptor superfamily member 9, *ACE* angiotensin converting enzyme, *AD-MSC* adipose tissue derived mesenchymal stem cells, *AgRP* Agouti-related protein, *ANG* angiotensin, *ANGPT* angiopoietin, *ANGPTL* angiopoietin like, *Anxa1* annexin A1, *AXL* tyrosine-protein kinase receptor UFO, *B2MG* beta-2-microglobulin, *BDNF* brain derived neurotrophic factor, *BLC* beta lymphocyte chemoattractant, *BM-MSCs* bone marrow derived mesenchymal stem cells, *BMP* bone morphogenetic protein, *BTC* probetacellulin, *BZ* border zone, *CCL* C–C motif chemokine, *CD* cluster of differentiation, *CFbs* cardiac fibroblasts, *CK β 8–1* C–C motif chemokine 23, *CMs* cardiomyocytes, *CNTF* ciliary neurotrophic factor, *COL* collagen, *CTACK* C–C motif chemokine 27, *CTF* cardiotrophin, *CSCs* cardiac stem cells, *CSF1* macrophage colony stimulating factor 1, *CTGF* cellular communication network family member 2, *CX3CL1* fractalkine, *CXCL* C-X-C motif chemokine, *DAG1* dystroglycan, *DCN* decorin, *DKK1* Dickkopf related protein 1, *dP/dT* contractility, *DTK* tyrosine-protein kinase receptor, *TYRO3EC* endothelial cell, *EDP* end diastolic pressure, *EGF* epidermal growth factor, *EGFR* epidermal growth factor receptor, *EMMPRIN* Basigin, *ENA78* C-X-C motif chemokine 5, *ENG* endoglin, *En-MSCs* menstrual blood derived mesenchymal stem cells, *EPCs* endothelial progenitor cells, *EPGN* epigen, *EPO* erythropoietin, *ET* endothelin, *Fas* tumour necrosis factor receptor superfamily member 6, *FGF* fibroblast growth factor, *Flt3* Receptor-type tyrosine-protein kinase, *FS* fractional shortening, *Gas6* growth arrest specific protein 6, *GCP2* C-X-C motif chemokine 6, *GCSF* granulocyte colony-stimulating factor, *GDF* growth/differentiation factor, *GDNF* glial cell line derived growth factor, *GFP* green fluorescent protein, *GITR* tumour necrosis factor receptor superfamily member 18, *GMCSF* granulocyte–macrophage colony-stimulating factor, *GRO* growth regulated, *GRN* progranulin, *GZMB* granzyme B, *HAI-1* Kunitz-type protease inhibitor 1, *HCC* C–C motif chemokine 16, *HGF* hepatocyte growth factor, *hDMECs* human dermal microvascular endothelial cells, *hMEC-1* human microvascular endothelial cells, *HIF* hypoxia inducible factor, *H/R* hypoxia/ reperfusion, *Hsp90b1* endoplasmin, *HUVECs* human umbilical vein endothelial cells, *I309* C–C motif chemokine 1, *ICAM* intercellular adhesion molecule 1, *IFN* interferon, *IGF* insulin like growth factor, *IGFBP* insulin like growth factor binding protein, *IL* interleukin, *INHBA* inhibin beta A chain, *I/R* ischemia/ reperfusion, *ITAC* C-X-C motif chemokine 11, *IZ* infarct zone, *KGF* fibroblast growth factor 7, *LDH* lactate dehydrogenase, *LEP* leptin, *LGALS1* galectin 1, *LG3BP* galectin-3 binding protein, *LIF* leukemia inhibitory factor, *LOC286987* hemiferrin, *LRP1* prolow-density lipoprotein receptor-related protein 1, *LVDD* left ventricular diastolic dysfunction, *LVDP* left ventricular developed pressure, *LVEDD* left ventricular end diastolic diameter, *LVEDP* left ventricular end diastolic pressure, *LVEDV* left ventricular end diastolic volume, *LVESV* left ventricular end systolic volume, *LVESD* left ventricular end systolic pressure, *LVEF* left ventricular ejection fraction, *LVIDD* left ventricular internal diameter end diastole, *LVISD* left ventricular internal diameter end systole, *LVSD* left ventricular systolic dysfunction, *LVSP* left ventricular systolic pressure, *MAdCAM* mucosal addressin cell adhesion molecule, *MCP* monocyte chemoattractant, *MCSF* macrophage colony-stimulating factor 1, *MDC* C–C motif chemokine 22, *MFGE8* lactadherin, *MIF* macrophage migration inhibitory factor, *MIG* macrophage induced gene, *MIP* macrophage inflammatory protein, *miR* microRNA, *MFGE8* lactadherin, *MIME* mimecan, *MMP* matrix metalloproteinase, *MRC2* c-type mannose receptor 2, *MSP* macrophage stimulating protein, *MYDGF* myeloid-derived growth factor, *NAP* neutrophil activating peptide, *NENF* neudesin, *NGF* beta nerve growth factor, *NRCs* neonatal rat cardiomyocytes, *NT* neurotrophin, *OPG* osteoprotegerin, *OPN* osteopontin, *PAI1* plasminogen activator inhibitor 1, *PARC* C–C motif chemokine 18, *PDGF* platelet derived growth factor, *PGE2* prostaglandin E2, *PIGF* phosphatidylinositol-glycan biosynthesis class F protein, *PLGF* placenta growth factor, *P-MSCs* placenta derived mesenchymal stem cells, *PTX* pentraxin, *RAGE* receptor for advanced glycosylation end products, *RANTES* C–C motif chemokine 5, *RVDP* right ventricular developed pressure, *RZ* remote zone, *SCF* stem cell factor, *Scg3* secretogranin 3, *SDF* stromal cell derived factor, *SFRP* secreted frizzled-related protein, *Sgp130* interleukin 6 receptor subunit beta, *sTNF R* soluble tumour necrosis factor receptor, *TACI* tumour necrosis factor receptor superfamily 13B, *Tagln* transgelin, *TARC* C–C motif chemokine 17, *TECK* C–C motif chemokine 25, *TGFβ* transforming growth factor beta, *TIMP* metalloproteinase inhibitor, *TNF* tumour necrosis factor, *Tpm* tropomyosin, *TRAIL R3* tumour necrosis factor receptor superfamily member 10c, *TRAIL R4* tumour necrosis factor receptor superfamily member 10c, *Tsp1* thrombospondin 1, *TWEAK* tumour necrosis factor ligand superfamily member 12, *TWEAKR* tumour necrosis factor receptor superfamily member 12A, *uPA* urokinase plasminogen activator, *uPAR* urokinase plasminogen activator surface receptor, *VCAM* vascular cell adhesion protein 1, *VEGF* vascular endothelial growth factor, *VEGFR* vascular endothelial growth factor receptor, *WISP2* cellular communication network family member 5, *XCL1* lymphotactin, *YKL40* Chitinase-3-like protein 1

## Discussion

In this systematic review, we have identified 234 factors that are directly released by MSCs. These factors potentially mediate improvements in cardiac outcomes in a paracrine fashion. Our review consolidates a considerable amount of evidence for the paracrine hypothesis, and demonstrates the potential beneficial effects of these factors in cardiac models of ischemia using a variety of in vitro, ex vivo, and in vivo experimental models. Furthermore, our quality assessment criteria enabled the identification of several aspects of study design that could be improved upon within the field.

The articles included in this study isolated MSCs from a broad range of sources derived from human, rat, mouse, or horse samples. These samples included bone marrow, cardiac tissue, adipose tissue, blood (peripheral, menstrual, and umbilical cord blood), and placenta. Investigators utilized a range of methods to identify the paracrine factors as detailed in Table 3, with the most common experimental approach being to culture the stem cells of interest for a few days and collect the supernatant or conditioned media of these cells. This conditioned media was then analyzed using experimental techniques such as ELISA, qPCR, western blot, immunostaining, mass spectrometry, immunoassays, and microarrays. Given the range of experimental methods used, comparisons made, controls used, and normalization approaches taken, we determined that it was not possible to quantitatively compare the available data. Thus we determined that the meta-analysis originally proposed in our PROSPERO submission would not be possible with the reported data. Rather, we provide a comprehensive list of the paracrine factors identified, without direct comparison between studies.

Quality assessment criteria are typically designed for evaluation of randomized clinical trials, and are thus unsuitable for evaluating in vitro studies that include a broad range of experimental design and methodologies. Therefore, we developed a 9-point checklist to assess the quality of reporting and overall design of the articles included in this systematic review. According to our quality assessment checklist only 11/86 studies were deemed to be of high quality (score of 80% or higher) including whether key aspects of study design such as cell passage or number, replicates, and appropriate controls were reported, or if the minimum criteria established by the ISCT [22] were met. Only one of the studies in this systematic review adhered completely to the set of standards proposed for identifying MSCs by the ISCT. Our quality assessment highlighted the fact that there is much variance in the methods used to derive and phenotype MSCs, the extent of reporting of these methods, as well as the approaches undertaken to identify released paracrine factors. Future studies should consider paying attention to the phenotyping profile recommended by the ISCT as a means of ensuring some level of standardization across the field, to promote reproducibility and reliability of acquired data. It would also be beneficial to consider adopting common nomenclature, and clearly reporting cell passage, the number of cells used therapeutically (whether in vitro, ex vivo, or in vivo), and sample size in order to prevent bias or the reporting of false positive results.

The factors identified in this study can be broadly classified as growth factors, cytokines, chemokines, hormones, enzymes, enzymatic inhibitors, receptors, or a range of protein classes including glycoproteins, binding proteins, and extracellular matrix proteins, amongst others (Fig. 2C). These factors have been implicated in functions such as angiogenesis, cytoprotection, and cell migration and proliferation [14, 16, 101]. Whilst the distinction was not specifically made in the studies included in this systematic review, it is important to acknowledge that the release of cargo from exosomes or extracellular vesicles could have unwittingly contributed to the quantified secretome. We found that MSCs or their conditioned media had anti-apoptotic, proliferative, and migratory effects on cardiomyocytes [1, 13, 15, 27, 29, 36, 38, 44, 47, 68, 70, 79, 97, 99] and endothelial cells (ECs) [13, 72, 85, 90, 91] under normoxic or hypoxic conditions in vitro. Furthermore MSCs or their conditioned media could induce tube formation in ECs [13, 15, 27, 51, 72, 85, 90, 91, 98], demonstrating their angiogenic properties.

Whilst studies have demonstrated that conditioned media of MSCs could be equally beneficial as whole cell therapy in various models of ischemic cardiac injury [10–13, 40], the manner in which whole cell therapy versus conditioned media therapy propagates its beneficial effects are likely to vary. MSCs delivered directly as a therapeutic option, would not only offload their cargo of paracrine factors, but further communicate with resident cardiac cells to promote further release of beneficial factors, or engage cell recruitment. For example the administration of cardiac adipose tissue derived MSCs induced a shift in macrophage phenotype from a pro-inflammatory M1 profile to an immunosuppressive and reparative M2 profile. This shift in macrophage polarization was also associated with changes to the profile of cytokine secretion [24]. Identifying means to control this shift could aid in the control and resolution of inflammation following a cardiac insult.

Further evidence for cellular crosstalk is available in in vitro studies where MSCs co-cultured with CMs induced changes in the secretion levels of various paracrine factors including VEGF, HGF, and SDF-1α [25]. Moreover, conditioned media collected from these co-cultures could enhance the protective effects of MSCs [25] and increase CM proliferation [68]. MSC co-culture with ECs promoted the formation of cell aggregation structures, which is indicative of their commitment to pre-vascularization, additionally co-culture resulted transcriptomic changes in MSCs and ECs, and altered their secretory profile of IL1β and IL6 [54].

Angoulvant et al. additionally compared the effects of MSCs that were freshly suspended in growth media to MSC conditioned media therapy, and demonstrated that freshly resuspended MSCs did not produce significant levels of growth factors, however they still afforded cardioprotection in an ex vivo model of I/R injury by reducing CM cell death. Thus suggesting that MSCs may be capable of protecting CMs via cell-to-cell communication or via secretion of growth factors once contact has been made with CMs [10].

These data suggest that the manner in which whole cell therapy versus conditioned media therapy modulates the micro-environment and facilitates cellular crosstalk, and thus further release of paracrine factors varies significantly. However, given the problems associated with whole cell therapy including cost, time to grow and deliver cells, donor matching, immune rejection, and the ethical and legal concerns associated with various stem cell types, using factors secreted by these cells instead may be a more logistically viable route. This would circumvent the traditional problems associated with whole cell therapy and provide a more readily accessible therapeutic product.

The most commonly identified factor, VEGF, was found in 62/81 of the included studies, and has been investigated extensively for its therapeutic effects in vitro and in vivo. It has been shown to play a role in improving cardiac function, reducing fibrosis, and promoting angiogenesis and cell proliferation [20, 35]. It is a central growth and survival factor in the injured heart [24, 33]; with Markel et al. demonstrated it is essential for BM-MSC mediated cardioprotection by inducing a VEGF knockdown [62]. However, in contrast, another study showed that culturing MSCs in the presence of VEGF neutralising antibodies, did not diminish the protective capacity of MSC conditioned media [10]. HGF was the second most abundantly identified protective factor (25/70), and is known to exert anti-apoptotic, pro-angiogenic, and pro-migratory effects on a range of cells. Moreover, when directly delivered in a rat model of MI resulted in improved cardiac function, and reduced infarct size [21, 38, 47]. Furthermore, a study in which endogenous HGF was neutralized and subsequently restored led to the attenuation of I/R injury and protected cardiomyocytes from cell death [102]. It seems likely that the protective effects of stem cell secretion are due to multiple secreted components, rather than one specific factor given these studies demonstrated cardioprotection despite targeted neutralization of VEGF and HGF, and that multiple potential beneficial factors were consistently identified across the studies included in this review, it seems likely that the protective effects of stem cell secretion are due to multiple secreted components and context dependent, rather than one specific factor being present irrespective of injury and timing.

It is worth noting that although this review included studies for identification of beneficial factors, two studies were identified which also determined that IL-1β and CXCL12 (also known as SDF1) were non-protective secreted factors [21, 103]. IL-1β is a cytokine that plays a key role in inflammatory processes in cardiac disease, it increases significantly in the myocardium in response to an acute ischemic event; and in the context of cardiac repair has contradictory implications. Toldo et al. demonstrated that anti IL-1β therapy in a mouse model of MI prevented deterioration of overall cardiac function and cardiomyocyte cell death [104]. Moreover, in the clinical CANTOS trial, targeting IL-1β with a therapeutic antibody, Canakinumab, significantly reduced high sensitivity C-reactive protein and IL-6 levels, and led to an overall reduction in rate of recurrence of cardiovascular events [105]. Thus suggesting that anti IL-1β therapy improves overall cardiovascular outcomes of MI patients. However, 6/81 included studies proposed IL-1β to be a potentially protective factor secreted by MSCs. This suggests that the effects of IL-1β are context (type of injury, timing, cellular-source) dependent. For example, Avolio et al. specifically determined that IL-1β is abundant in the secretome of CSCs isolated from failing hearts, and has no anti-apoptotic effects in an in vitro model of I/R. Whereas CSCs derived from healthy donor hearts did [103]. They further determined that pre-incubation of failing heart CSCs with an IL-1β neutralising antibody could restore their anti-apoptotic properties. Thus demonstrating that IL-1β secretion by failing heart CSCs abolishes the protective effects of the CSC secretome. CXCL12/SDF-1 is a chemokine implicated in cardiogenesis, and recruitment of endothelial progenitor cells and other stem cells to sites of ischemic damage [3, 21]. Although we identified one study that suggested CXCL12/SDF-1 to be non-protective, the majority of articles included in the present study (18/81) identified CXCL12/SDF-1 as a potentially beneficial factor secreted by MSCs. For example, Huang et al. demonstrated that downregulating SDF-1 expression in CSCs completely abolished the beneficial effects of CSCs on cardiac function. Furthermore, blocking the SDF-1 receptor in the heart significantly attenuated the beneficial effects of CSCs in an ex vivo model of I/R injury [3]. Thus demonstrating that SDF-1 is a key factor via which this particular population of CSCs exert their effects.

The functional benefits of MSC therapy ex vivo or in vivo were investigated in 52/81 of the included studies. The dominant model used in ex vivo studies was the Langendorff based I/R injury model. These studies identified HGF, IGF-1, IL-10, TNFα, SDF-1, and VEGF as being secreted by BM-MSCs [3, 10, 33, 40, 41, 43, 48, 60, 62, 73] or CSCs [3] in their conditioned media. The majority of these studies perfused BM-MSCs or CSCs as whole cell therapy [3, 33, 40, 41, 43, 48, 60, 62, 73]. The improvements in infarct size and cardiac function reported in each were subsequently attributed to the paracrine factors released by MSCs, although causative data was not always present. Two studies, however, did investigate a causal link by perfusing the conditioned media of BM-MSCs [10] or CSCs [3] in their experimental model. The first demonstrated that the conditioned media of BM-MSCs was equally effective at reducing cardiac injury as BM-MSCs in both in vitro and ex vivo simulated ischemia models [10]. Furthermore, Huang et al., identified SDF-1 as being a highly abundant paracrine factor secreted by CSCs. They determined that the paracrine factors of CSCs mediated cardioprotection when delivered pre-I/R [3]. Whilst it is important to note that ex vivo experimental methods cannot recapitulate the recruitment of various cell types including immune cells to the heart and investigate their dynamic interaction; experiments utilizing conditioned media are able to test a causal relationship between the factors released by MSCs and observed improvements in cardiac outcomes.

Similar patterns were present in the in vivo experiments conducted within the included articles. Investigators commonly injected MSCs intramuscularly or intravenously at varying periods following permanent or transient induction of MI. Conditioned media was only delivered in four of the included studies utilizing MI models [13, 15, 29, 79]. These studies demonstrated that the conditioned media of MSCs derived from adipose tissue [29], bone marrow [13, 79], and placenta [15], could protect CMs from cell death under hypoxic conditions [13, 15, 29, 79]. Furthermore, utilizing the conditioned media therapeutically in in vivo models of MI improved systolic and diastolic function, reduced overall infarct size, prevented cell death in the infarcted area, and increased vessel density when compared to control media [13, 15, 29, 79]. The reported improvements in cardiac outcomes present in these studies provide evidence for the paracrine hypothesis, and suggests that the factors released by MSCs could potentially be equally beneficial therapeutic options.

Anderson et al., took this premise a step further and trialled specific factors identified in vitro in a LAD model of I/R. They found that HGF, but not CXCL2, soaked micro-sponges could significantly reduce infarct size, improve cardiac function, and prevent CM apoptosis [21]. In line with these findings, Yeghiazarians et al. reported that delivery of bone marrow cell extract 3 days post MI, reduced infarct size and improved overall cardiac function and vessel density to a comparable extent to whole cell therapy [12]. A follow up from Yeghiazarians et al. demonstrated that IL-15, a factor identified as being highly expressed in the bone marrow cell extract, could protect CMs from cell death and oxidative stress under hypoxia in vitro [11]. Furthermore, they demonstrated that IL-15 can be protective in a model of mouse MI, by improving cardiac function, and reducing infarct size and CM cell death [106]. A study by Angeli et al., demonstrated that the administration of the cell extracts of human mononuclear cells and bone marrow cells 2 days post-MI in mice resulted in a significant increase in LVEF, vascular density at the border zone, and reduced infarct size [107]. In line with these findings, the data present in the included studies further demonstrate that the intact cell may not be essential to achieve cardiac repair.

In conclusion, this systematic review has enabled the identification and consolidation of 228 individual factors known to be secreted by MSCs, which may have protective effects in cardiac models of ischemia. In the included studies, a significant number investigated the effects of MSC therapy in vivo or ex vivo. Of particular interest were those that clearly demonstrated that treatment with either the conditioned media of MSCs or the factors identified within promote effects which are equally beneficial as whole cell therapy. Together these studies suggest that the release of soluble, pro-reparative factors by transplanted MSCs are responsible for the beneficial effects reported, providing strong support for the paracrine hypothesis of cardiac repair. The factors released by MSCs have significant potential to lead to the identification of novel therapeutic targets, thus making way for alternative and more effective therapeutic options for treating cardiac fibrosis and heart failure which could drastically improve the health outcomes of patients suffering from CVDs.

## Supplementary Information

Below is the link to the electronic supplementary material.

Supplementary file1 (DOCX 64 kb)

## Data Availability

The datasets generated during and/or analysed during the current study are available from the corresponding author on reasonable request.
