# Supplementary material for: Paracrine Factors Released by Stem Cells of Mesenchymal Origin and their Effects in Cardiovascular Disease: A Systematic Review of Pre-clinical Studies
Source: Stem Cell Rev Rep. 2022 Jul 28;18(8):2606–28. doi: 10.1007/s12015-022-10429-6 (PMC9622561; doi:10.1007/s12015-022-10429-6)
Supplement: Supplementary file 1 — Supplementary file1 (DOCX 64 kb) [file 12015_2022_10429_MOESM1_ESM.docx]

**Supplemental Information**

**Table 1.** Search strategy for the Embase database

| Number | Search Terms |
| --- | --- |
| 1 | Mesenchymal stem cells/ |
| 2 | Bone marrow derived mesenchymal stem cell/ |
| 3 | Bone marrow stoma cell/ |
| 4 | Bone marrow stomal cell/ |
| 5 | ("Mesenchymal stem cell*" or "bone marrow derived mesenchymal stem cell*" or "bone marrow stroma cells*" or "bone marrow stromal cell*").ti,ab,kw. |
| 6 | (("Cardiac" or "endothelial" or "myoblast" or "fibroblast") adj "progenitor cell*").mp. [mp=title, abstract, heading word, drug trade name, original title, device manufacturer, drug manufacturer, device trade name, keyword, floating subheading word, candidate term word] |
| 7 | Myocardial ischemia/ |
| 8 | Acute coronary syndrome/ |
| 9 | Myocardial infarction/ |
| 10 | ST elevation myocardial infarction/ |
| 11 | Non-ST elevated myocardial infarction/ |
| 12 | Ischemic heart disease/ |
| 13 | Heart infarction/ |
| 14 | Acute heart infarction/ |
| 15 | Heart muscle ischemia/ |
| 16 | ("Myocardial ischemia*" or "acute coronary syndrome*" or "myocardial infarction*" or "heart infarction*" or "ST-elevation myocardial infarction*" or "non-ST-segment elevation myocardial infarction*" or "heart muscle ischemia*" or "ischemic heart disease*" or STEMI* or NSTEMI* or MI* or IHD*).ti,ab,kw. |
| 17 | ("Cardiac" or "heart" or "myocard*").mp. |
| 18 | Paracrine signaling/ |
| 19 | Cellular secretion/ |
| 20 | ("Paracrine signalling*" or "cellular secretion*" or "paracrine effect*" or "paracrine communication*").ti,ab,kw. |
| 21 | 1 or 2 or 3 or 4 or 5 or 6 |
| 22 | 7 or 8 or 9 or 10 or 11 or 12 or 13 or 14 or 15 or 16 or 17 |
| 23 | 18 or 19 or 20 |
| 24 | 21 and 22 and 23 |
| 25 | Limit 24 to English language |

**Table 2.** Search strategy for the Medline database

| Number | Search Term |
| --- | --- |
| 1 | Mesenchymal stem cells/ |
| 2 | (Mesenchymal stromal cell* or stem cell*).mp. [mp=title, abstract, original title, name of substance word, subject heading word, floating sub-heading word, keyword heading word, organism supplementary concept word, protocol supplementary concept word, rare disease supplementary concept word, unique identifier, synonyms] |
| 3 | Mesenchymal stroma cell*.mp. |
| 4 | (Mesenchymal adj (progenitor cell* or adult progenitor cell*)).mp. [mp=title, abstract, original title, name of substance word, subject heading word, floating sub-heading word, keyword heading word, organism supplementary concept word, protocol supplementary concept word, rare disease supplementary concept word, unique identifier, synonyms] |
| 5 | ((Cardiac or endothelial or myoblast or fibroblast) adj progenitor cell*).mp. [mp=title, abstract, original title, name of substance word, subject heading word, floating sub-heading word, keyword heading word, organism supplementary concept word, protocol supplementary concept word, rare disease supplementary concept word, unique identifier, synonyms] |
| 6 | 1 or 2 or 3 or 4 or 5 |
| 7 | Myocardial ischemia/ |
| 8 | Acute coronary syndrome/ |
| 9 | Myocardial infarction/ |
| 10 | ST elevation myocardial infarction/ |
| 11 | Non-ST elevated myocardial infarction/ |
| 12 | (Cardiac or heart or myocard*).mp. [mp=title, abstract, original title, name of substance word, subject heading word, floating sub-heading word, keyword heading word, organism supplementary concept word, protocol supplementary concept word, rare disease supplementary concept word, unique identifier, synonyms] |
| 13 | 7 or 8 or 9 or 10 or 11 or 12 |
| 14 | Paracrine communication/ |
| 15 | (Paracrine or release* or secret*).mp. [mp=title, abstract, original title, name of substance word, subject heading word, floating sub-heading word, keyword heading word, organism supplementary concept word, protocol supplementary concept word, rare disease supplementary concept word, unique identifier, synonyms] |
| 16 | 14 or 15 |
| 17 | 6 and 13 and 16 |
| 18 | Limit 17 to English language |

**Table 3.** Quality assessment scores as determined by two independent reviewers

|  |  | **Assessed by N.S.M.** | | | | | | | | | **Assessed by L.R.** | | | | | | | | | **N.M** | **L.R** |  |  |
| --- | --- | --- | --- | --- | --- | --- | --- | --- | --- | --- | --- | --- | --- | --- | --- | --- | --- | --- | --- | --- | --- | --- | --- |
| **First Author** | **Year** | **QA1** | **QA2** | **QA3** | **QA4** | **QA5** | **QA6** | **QA7** | **QA8** | **QA9** | **QA1** | **QA2** | **QA3** | **QA4** | **QA5** | **QA6** | **QA7** | **QA8** | **QA9** | **Score (/17)** | | **Mean Score (/17)** | **Percentage (%)** |
| Abarbanell | 2010 | 2 | 2 | 2 | 2 | 1 | 0 | 2 | 2 | 1 | 2 | 2 | 2 | 2 | 1 | 0 | 2 | 2 | 1 | 14 | 14 | 14 | 82.35 |
| Adutler-Lieber | 2013 | 2 | 2 | 2 | 1 | 1 | 0 | 0 | 1 | 1 | 2 | 2 | 2 | 1 | 1 | 0 | 0 | 1 | 1 | 10 | 10 | 10 | 58.82 |
| Alijani-Ghazyani | 2021 | 1 | 2 | 1 | 1 | 1 | 1 | 0 | 2 | 1 | 1 | 2 | 1 | 0 | 0 | 0 | 0 | 2 | 1 | 10 | 7 | 8.5 | 50.00 |
| Alrefai | 2019 | 2 | 1 | 2 | 1 | 0 | 1 | 0 | 1 | 1 | 1 | 1 | 2 | 1 | 0 | 1 | 0 | 1 | 1 | 9 | 8 | 8.5 | 50.00 |
| Anderson | 2008 | 2 | 2 | 2 | 1 | 1 | 1 | 0 | 2 | 1 | 2 | 2 | 2 | 1 | 1 | 1 | 0 | 1 | 1 | 12 | 11 | 11.5 | 67.65 |
| Angoulvant | 2011 | 2 | 2 | 2 | 1 | 1 | 2 | 2 | 2 | 1 | 2 | 2 | 2 | 1 | 1 | 2 | 2 | 2 | 1 | 15 | 15 | 15 | 88.24 |
| Augustin | 2013 | 1 | 2 | 2 | 1 | 0 | 0 | 0 | 1 | 1 | 1 | 1 | 2 | 1 | 0 | 0 | 0 | 1 | 1 | 8 | 7 | 7.5 | 44.12 |
| Avolio | 2015 | 2 | 2 | 2 | 1 | 1 | 1 | 0 | 2 | 1 | 2 | 2 | 2 | 1 | 1 | 0 | 0 | 1 | 1 | 12 | 10 | 11 | 64.71 |
| Bader | 2014 | 2 | 2 | 2 | 1 | 1 | 2 | 2 | 2 | 1 | 2 | 2 | 2 | 1 | 1 | 2 | 2 | 1 | 1 | 15 | 14 | 14.5 | 85.29 |
| Baffour | 2006 | 1 | 2 | 2 | 1 | 1 | 2 | 1 | 2 | 1 | 1 | 2 | 2 | 1 | 1 | 1 | 1 | 2 | 1 | 13 | 12 | 12.5 | 73.53 |
| Bao | 2017 | 2 | 2 | 2 | 2 | 1 | 1 | 1 | 1 | 1 | 1 | 0 | 2 | 2 | 1 | 2 | 2 | 2 | 1 | 13 | 13 | 13 | 76.47 |
| Bayes-Genis | 2010 | 0 | 1 | 2 | 1 | 1 | 1 | 2 | 1 | 1 | 0 | 1 | 2 | 1 | 1 | 1 | 2 | 1 | 0 | 10 | 9 | 9.5 | 55.88 |
| Burlacu | 2013 | 2 | 2 | 2 | 1 | 1 | 0 | 1 | 2 | 1 | 2 | 0 | 2 | 1 | 1 | 0 | 2 | 2 | 1 | 12 | 11 | 11.5 | 67.65 |
| Bussche | 2014 | 2 | 2 | 2 | 1 | 1 | 2 | 1 | 2 | 1 | 2 | 2 | 2 | 1 | 1 | 0 | 1 | 2 | 1 | 14 | 12 | 13 | 76.47 |
| Cai | 2019 | 2 | 2 | 2 | 2 | 1 | 1 | 1 | 1 | 1 | 2 | 2 | 2 | 2 | 1 | 0 | 1 | 2 | 1 | 13 | 13 | 13 | 76.47 |
| Chen | 2014 | 1 | 2 | 2 | 1 | 1 | 0 | 0 | 2 | 1 | 2 | 2 | 2 | 1 | 1 | 1 | 0 | 1 | 1 | 10 | 11 | 10.5 | 61.76 |
| Constantinou | 2020 | 2 | 2 | 1 | 2 | 1 | 0 | 1 | 2 | 1 | 2 | 2 | 1 | 2 | 1 | 0 | 1 | 2 | 1 | 12 | 12 | 12 | 70.59 |
| Crisostomo | 2008 | 2 | 2 | 2 | 1 | 1 | 1 | 2 | 2 | 1 | 2 | 2 | 2 | 2 | 1 | 2 | 1 | 2 | 1 | 14 | 15 | 14.5 | 85.29 |
| Crisostomo | 2007 | 2 | 2 | 2 | 2 | 1 | 2 | 2 | 2 | 1 | 2 | 2 | 2 | 2 | 1 | 2 | 2 | 2 | 1 | 16 | 16 | 16 | 94.12 |
| Crisostomo | 2007 | 2 | 2 | 2 | 2 | 1 | 2 | 1 | 2 | 1 | 1 | 2 | 1 | 2 | 1 | 2 | 2 | 2 | 1 | 15 | 14 | 14.5 | 85.29 |
| Crisostomo | 2006 | 1 | 1 | 2 | 2 | 1 | 2 | 2 | 2 | 1 | 2 | 2 | 2 | 2 | 1 | 2 | 2 | 2 | 1 | 14 | 16 | 15 | 88.24 |
| Crisostomo | 2019 | 2 | 2 | 2 | 2 | 1 | 2 | 1 | 1 | 1 | 1 | 2 | 2 | 2 | 1 | 2 | 1 | 1 | 1 | 14 | 13 | 13.5 | 79.41 |
| Cui | 2016 | 2 | 2 | 2 | 1 | 1 | 2 | 1 | 2 | 1 | 1 | 2 | 2 | 1 | 1 | 2 | 1 | 1 | 1 | 14 | 12 | 13 | 76.47 |
| Czapla | 2016 | 1 | 2 | 2 | 1 | 1 | 1 | 0 | 1 | 1 | 1 | 1 | 2 | 1 | 1 | 0 | 0 | 1 | 1 | 10 | 8 | 9 | 52.94 |
| Dai | 2007 | 2 | 2 | 2 | 1 | 1 | 2 | 0 | 2 | 1 | 2 | 2 | 2 | 1 | 1 | 2 | 0 | 2 | 1 | 13 | 13 | 13 | 76.47 |
| Daltro | 2017 | 1 | 2 | 2 | 2 | 1 | 0 | 1 | 2 | 1 | 1 | 1 | 2 | 2 | 1 | 0 | 1 | 0 | 1 | 12 | 9 | 10.5 | 61.76 |
| Danieli | 2015 | 2 | 1 | 2 | 1 | 1 | 2 | 0 | 2 | 1 | 2 | 2 | 2 | 1 | 1 | 0 | 0 | 2 | 1 | 12 | 11 | 11.5 | 67.65 |
| Deng | 2020 | 2 | 2 | 1 | 1 | 1 | 2 | 0 | 1 | 1 | 2 | 2 | 1 | 1 | 1 | 2 | 0 | 1 | 1 | 11 | 11 | 11 | 64.71 |
| Deuse | 2010 | 2 | 2 | 1 | 1 | 1 | 1 | 1 | 2 | 1 | 1 | 1 | 2 | 1 | 1 | 0 | 0 | 1 | 1 | 12 | 8 | 10 | 58.82 |
| Duran | 2013 | 1 | 1 | 2 | 2 | 1 | 1 | 1 | 1 | 1 | 1 | 1 | 2 | 2 | 1 | 0 | 1 | 2 | 1 | 11 | 11 | 11 | 64.71 |
| Erwin | 2009 | 2 | 2 | 2 | 2 | 1 | 0 | 1 | 1 | 1 | 2 | 2 | 2 | 2 | 1 | 0 | 1 | 0 | 1 | 12 | 11 | 11.5 | 67.65 |
| Fan | 2015 | 2 | 1 | 1 | 2 | 1 | 2 | 0 | 1 | 1 | 2 | 2 | 2 | 2 | 1 | 2 | 0 | 1 | 1 | 11 | 13 | 12 | 70.59 |
| Fan | 2009 | 1 | 2 | 2 | 2 | 1 | 2 | 2 | 2 | 1 | 2 | 2 | 2 | 2 | 1 | 2 | 2 | 2 | 1 | 15 | 16 | 15.5 | 91.18 |
| Fanton | 2016 | 2 | 2 | 2 | 1 | 0 | 1 | 1 | 1 | 1 | 2 | 2 | 2 | 1 | 0 | 1 | 1 | 2 | 1 | 11 | 12 | 11.5 | 67.65 |
| Figeac | 2014 | 2 | 2 | 2 | 1 | 0 | 0 | 0 | 2 | 1 | 2 | 2 | 2 | 1 | 0 | 0 | 0 | 2 | 1 | 10 | 10 | 10 | 58.82 |
| Huang | 2013 | 1 | 2 | 2 | 1 | 1 | 0 | 1 | 1 | 1 | 2 | 2 | 2 | 1 | 1 | 0 | 1 | 1 | 1 | 10 | 11 | 10.5 | 61.76 |
| Huang | 2013 | 1 | 1 | 2 | 2 | 1 | 2 | 0 | 1 | 1 | 1 | 1 | 1 | 2 | 1 | 0 | 0 | 1 | 1 | 11 | 8 | 9.5 | 55.88 |
| Huang | 2011 | 1 | 1 | 2 | 1 | 1 | 1 | 1 | 1 | 1 | 1 | 1 | 1 | 1 | 1 | 1 | 0 | 1 | 1 | 10 | 8 | 9 | 52.94 |
| Iso | 2007 | 1 | 1 | 2 | 0 | 0 | 1 | 1 | 1 | 1 | 1 | 1 | 1 | 1 | 0 | 1 | 1 | 1 | 1 | 8 | 8 | 8 | 47.06 |
| Jiang | 2013 | 1 | 1 | 1 | 1 | 1 | 0 | 1 | 1 | 1 | 1 | 1 | 2 | 1 | 1 | 0 | 1 | 1 | 1 | 8 | 9 | 8.5 | 50.00 |
| Ju | 2018 | 1 | 1 | 1 | 1 | 1 | 1 | 0 | 1 | 1 | 1 | 1 | 1 | 1 | 1 | 0 | 0 | 1 | 1 | 8 | 7 | 7.5 | 44.12 |
| Latham | 2013 | 1 | 1 | 2 | 1 | 0 | 1 | 0 | 1 | 1 | 1 | 1 | 2 | 1 | 0 | 0 | 0 | 1 | 1 | 8 | 7 | 7.5 | 44.12 |
| Li | 2012 | 0 | 2 | 2 | 1 | 1 | 1 | 1 | 1 | 1 | 1 | 2 | 1 | 1 | 1 | 0 | 1 | 1 | 1 | 10 | 9 | 9.5 | 55.88 |
| Li | 2015 | 2 | 1 | 2 | 0 | 1 | 0 | 1 | 1 | 1 | 1 | 1 | 2 | 0 | 1 | 0 | 1 | 1 | 1 | 9 | 8 | 8.5 | 50.00 |
| Li | 2020 | 2 | 2 | 2 | 2 | 1 | 1 | 1 | 2 | 1 | 2 | 2 | 2 | 2 | 1 | 1 | 1 | 2 | 1 | 14 | 14 | 14 | 82.35 |
| Li | 2020 | 1 | 1 | 2 | 1 | 0 | 2 | 0 | 2 | 1 | 1 | 1 | 2 | 1 | 0 | 2 | 0 | 2 | 1 | 10 | 10 | 10 | 58.82 |
| Li | 2020 | 2 | 1 | 2 | 0 | 0 | 0 | 0 | 1 | 1 | 1 | 1 | 2 | 0 | 0 | 0 | 0 | 1 | 1 | 7 | 6 | 6.5 | 38.24 |
| Li | 2017 | 1 | 2 | 2 | 2 | 0 | 0 | 1 | 0 | 1 | 2 | 2 | 2 | 2 | 0 | 0 | 1 | 0 | 1 | 9 | 10 | 9.5 | 55.88 |
| Li | 2021 | 2 | 1 | 2 | 1 | 1 | 0 | 1 | 2 | 1 | 2 | 2 | 2 | 1 | 1 | 0 | 1 | 2 | 1 | 11 | 12 | 11.5 | 67.65 |
| Lin | 2020 | 2 | 2 | 2 | 1 | 1 | 2 | 1 | 1 | 1 | 2 | 2 | 2 | 1 | 1 | 2 | 1 | 1 | 1 | 13 | 13 | 13 | 76.47 |
| Liu | 2014 | 1 | 2 | 2 | 1 | 0 | 0 | 1 | 0 | 1 | 2 | 2 | 2 | 1 | 0 | 0 | 1 | 0 | 1 | 8 | 9 | 8.5 | 50.00 |
| Lu | 2013 | 1 | 1 | 1 | 1 | 1 | 1 | 1 | 0 | 1 | 1 | 1 | 1 | 1 | 1 | 0 | 1 | 0 | 1 | 8 | 7 | 7.5 | 44.12 |
| Luo | 2012 | 2 | 2 | 2 | 2 | 1 | 1 | 1 | 0 | 1 | 2 | 2 | 2 | 2 | 1 | 1 | 1 | 0 | 1 | 12 | 12 | 12 | 70.59 |
| Mao | 2019 | 2 | 1 | 1 | 1 | 1 | 0 | 0 | 0 | 1 | 2 | 1 | 1 | 1 | 1 | 0 | 0 | 0 | 1 | 7 | 7 | 7 | 41.18 |
| Markel | 2008 | 1 | 1 | 2 | 1 | 1 | 1 | 1 | 1 | 1 | 1 | 1 | 2 | 1 | 1 | 1 | 1 | 1 | 1 | 10 | 10 | 10 | 58.82 |
| McQuaig | 2020 | 2 | 2 | 2 | 2 | 1 | 2 | 1 | 2 | 1 | 2 | 2 | 2 | 2 | 1 | 2 | 1 | 2 | 1 | 15 | 15 | 15 | 88.24 |
| Meng | 2018 | 1 | 2 | 2 | 1 | 1 | 0 | 0 | 1 | 1 | 1 | 2 | 2 | 1 | 1 | 0 | 0 | 1 | 1 | 9 | 9 | 9 | 52.94 |
| Montzka | 2010 | 1 | 1 | 2 | 1 | 2 | 1 | 1 | 0 | 1 | 1 | 1 | 2 | 1 | 2 | 0 | 0 | 0 | 1 | 10 | 8 | 9 | 52.94 |
| Nakanishi | 2011 | 1 | 2 | 2 | 2 | 1 | 0 | 1 | 1 | 1 | 2 | 2 | 2 | 2 | 1 | 0 | 2 | 1 | 1 | 11 | 13 | 12 | 70.59 |
| Page | 2014 | 2 | 2 | 2 | 1 | 0 | 0 | 0 | 1 | 1 | 2 | 2 | 2 | 1 | 0 | 0 | 0 | 1 | 1 | 9 | 9 | 9 | 52.94 |
| Paquet | 2015 | 2 | 2 | 2 | 1 | 0 | 1 | 1 | 1 | 1 | 2 | 2 | 2 | 1 | 0 | 2 | 1 | 1 | 1 | 11 | 12 | 11.5 | 67.65 |
| Popescu | 2021 | 1 | 2 | 1 | 1 | 1 | 0 | 2 | 2 | 1 | 1 | 2 | 2 | 1 | 0 | 0 | 2 | 2 | 1 | 11 | 11 | 11 | 64.71 |
| RanjendranNair | 2017 | 1 | 2 | 2 | 1 | 1 | 1 | 2 | 1 | 1 | 2 | 1 | 2 | 1 | 1 | 2 | 1 | 1 | 1 | 12 | 12 | 12 | 70.59 |
| Sadat | 2007 | 2 | 2 | 2 | 1 | 1 | 0 | 0 | 1 | 1 | 2 | 2 | 2 | 1 | 1 | 0 | 1 | 1 | 1 | 10 | 11 | 10.5 | 61.76 |
| Samal | 2019 | 2 | 2 | 2 | 1 | 0 | 0 | 1 | 1 | 1 | 2 | 2 | 2 | 1 | 0 | 0 | 1 | 1 | 1 | 10 | 10 | 10 | 58.82 |
| Sassoli | 2011 | 1 | 2 | 2 | 2 | 1 | 2 | 2 | 1 | 1 | 2 | 2 | 2 | 2 | 1 | 2 | 2 | 1 | 1 | 14 | 15 | 14.5 | 85.29 |
| See | 2011 | 2 | 1 | 2 | 1 | 1 | 0 | 1 | 2 | 1 | 2 | 1 | 2 | 1 | 1 | 0 | 1 | 2 | 1 | 11 | 11 | 11 | 64.71 |
| Shan | 2018 | 2 | 2 | 2 | 2 | 1 | 0 | 0 | 1 | 1 | 2 | 2 | 2 | 2 | 1 | 0 | 0 | 1 | 1 | 11 | 11 | 11 | 64.71 |
| Song | 2016 | 2 | 1 | 1 | 1 | 0 | 0 | 1 | 1 | 1 | 2 | 0 | 1 | 1 | 0 | 0 | 0 | 1 | 1 | 8 | 6 | 7 | 41.18 |
| Song | 2017 | 2 | 2 | 2 | 1 | 0 | 1 | 1 | 1 | 1 | 2 | 2 | 2 | 2 | 0 | 0 | 1 | 1 | 1 | 11 | 11 | 11 | 64.71 |
| Tang | 2011 | 1 | 2 | 2 | 1 | 1 | 1 | 1 | 2 | 1 | 2 | 2 | 2 | 1 | 1 | 1 | 1 | 2 | 1 | 12 | 13 | 12.5 | 73.53 |
| Thej | 2017 | 1 | 2 | 2 | 1 | 1 | 2 | 1 | 2 | 1 | 2 | 2 | 2 | 1 | 1 | 2 | 1 | 2 | 1 | 13 | 14 | 13.5 | 79.41 |
| Wairiuko | 2007 | 1 | 1 | 1 | 1 | 1 | 1 | 1 | 1 | 1 | 0 | 0 | 1 | 1 | 1 | 0 | 1 | 1 | 1 | 9 | 6 | 7.5 | 44.12 |
| Wang | 2014 | 2 | 2 | 2 | 2 | 1 | 0 | 2 | 1 | 1 | 2 | 2 | 2 | 2 | 1 | 0 | 1 | 1 | 1 | 13 | 12 | 12.5 | 73.53 |
| Windmolders | 2014 | 2 | 2 | 2 | 1 | 1 | 0 | 1 | 1 | 1 | 2 | 2 | 2 | 1 | 1 | 0 | 1 | 1 | 1 | 11 | 11 | 11 | 64.71 |
| Xia | 2015 | 1 | 1 | 2 | 2 | 1 | 2 | 1 | 1 | 1 | 2 | 1 | 2 | 2 | 1 | 2 | 1 | 1 | 1 | 12 | 13 | 12.5 | 73.53 |
| Xu | 2007 | 2 | 1 | 1 | 1 | 1 | 1 | 2 | 1 | 1 | 2 | 1 | 1 | 1 | 1 | 0 | 2 | 1 | 1 | 11 | 10 | 10.5 | 61.76 |
| Yan | 2020 | 2 | 2 | 1 | 2 | 1 | 2 | 1 | 1 | 1 | 2 | 2 | 1 | 2 | 1 | 2 | 1 | 1 | 1 | 13 | 13 | 13 | 76.47 |
| Yang | 2012 | 2 | 2 | 2 | 1 | 1 | 1 | 2 | 2 | 1 | 2 | 2 | 1 | 1 | 1 | 1 | 2 | 2 | 1 | 14 | 13 | 13.5 | 79.41 |
| Yang | 2021 | 1 | 2 | 2 | 1 | 0 | 0 | 2 | 2 | 1 | 2 | 2 | 2 | 1 | 0 | 0 | 2 | 2 | 1 | 11 | 12 | 11.5 | 67.65 |
| Yu | 2009 | 1 | 2 | 2 | 1 | 1 | 0 | 0 | 1 | 1 | 1 | 2 | 1 | 1 | 1 | 0 | 0 | 1 | 1 | 9 | 8 | 8.5 | 50.00 |
| Zeng | 2008 | 1 | 2 | 2 | 2 | 1 | 2 | 2 | 1 | 1 | 2 | 1 | 1 | 2 | 1 | 2 | 0 | 1 | 0 | 14 | 10 | 12 | 70.59 |
| Zhang | 2007 | 1 | 2 | 2 | 1 | 1 | 1 | 1 | 1 | 1 | 2 | 2 | 2 | 1 | 1 | 1 | 2 | 1 | 1 | 11 | 13 | 12 | 70.59 |
| Zhang | 2015 | 1 | 1 | 2 | 2 | 1 | 0 | 0 | 1 | 1 | 1 | 1 | 2 | 2 | 1 | 0 | 0 | 0 | 1 | 9 | 8 | 8.5 | 50.00 |
| Zhao | 2020 | 1 | 2 | 1 | 1 | 1 | 0 | 1 | 2 | 1 | 1 | 1 | 1 | 1 | 1 | 0 | 1 | 2 | 1 | 10 | 9 | 9.5 | 55.88 |
| Zhou | 2021 | 1 | 2 | 2 | 2 | 0 | 1 | 1 | 2 | 1 | 2 | 2 | 2 | 2 | 0 | 1 | 1 | 2 | 1 | 12 | 13 | 12.5 | 73.53 |
